# Efficient material-induced activation of monocyte-derived dendritic cells releasing surface molecules, matrix metalloproteinases, and growth factors needed for regenerative tissue remodeling

**DOI:** 10.1016/j.mtbio.2023.100869

**Published:** 2023-11-15

**Authors:** Daniel David Stöbener, Andrea Cosimi, Marie Weinhart, Matthias Peiser

**Affiliations:** a Institute of Chemistry and Biochemistry - Organic Chemistry, Freie Universität Berlin, Takustr. 3, 14195, Berlin, Germany; b Institute of Physical Chemistry and Electrochemistry, Leibniz Universität Hannover, Callinstr. 3A, 30167, Hannover, Germany; c Institute of Chemistry and Biochemistry - Biochemistry, Freie Universität Berlin, Thielallee 63, 14195, Berlin, Germany

**Keywords:** Immunoengineering, Functional surface coatings, Poly(glycidyl ether) copolymers, *Ex vivo* dendritic cell priming, Regenerative medicine

## Abstract

New experimental approaches for tissue repair have recently been proposed and include the application of natural or synthetic biomaterials and immune cells. Herein, fully synthetic poly(glycidyl ether) (PGE) copolymer coatings are evaluated as bioinstructive materials for the *in vitro* culture and intrinsic activation of human immune cells. Immature monocyte-derived dendritic cells (moDCs) are exposed to PGE brush and gel coatings of varying copolymer composition, wettability, and deformability immobilized on polystyrene culture dishes. Compared to moDCs cultured on standard tissue culture-treated polystyrene, activation marker levels on the cell surface are strongly enhanced on PGE substrates. Thereby, moDCs undergo a distinct morphological change and reach levels of activation comparable to those achieved by toll-like receptor (TLR) ligand liposaccharide (LPS), specifically for the expression of costimulatory molecules CD86 and CD40 as well as human leukocyte antigen (HLA)-DR. In addition, PGE coatings induce a significantly enhanced level of programmed cell death ligands 1 and 2 (PD-L1/-L2) on the moDC surface, two molecules crucially involved in maintaining immune tolerance. In addition, an increased release of matrix metalloproteinases MMP-1 and MMP-7, as well as transforming growth factor (TGF)-β1 and epidermal growth factor (EGF) was observed in moDCs cultured on PGE substrates. As fully synthetic biomaterials, PGE coatings demonstrate intrinsic functional competence in instructing immature human moDCs for phenotypic activation *in vitro,* accompanied by the secretion of bioactive molecules, which are known to be crucial for tissue regeneration. Hence, PGE coatings hold strong potential for immune-modulating implant coatings, while PGE-activated moDCs are promising candidates for future clinical cell-based immunoengineering therapies.

## Highlights

• PGE-coated polystyrene culture substrates activate immature human DCs *in vitro*.
• DC surface marker expression is enhanced to a degree typically only found *in vivo*.
• DCs secrete considerable amounts of regenerative growth factors TGF-β and EGF.
• DC tolerogenicity suggests utility of tissue-regenerative PGE implant coatings.
• *In vitro*-activated DCs bear high potential for *in vivo* cell-based immunotherapies.

## Graphical abstract

**Figure:**
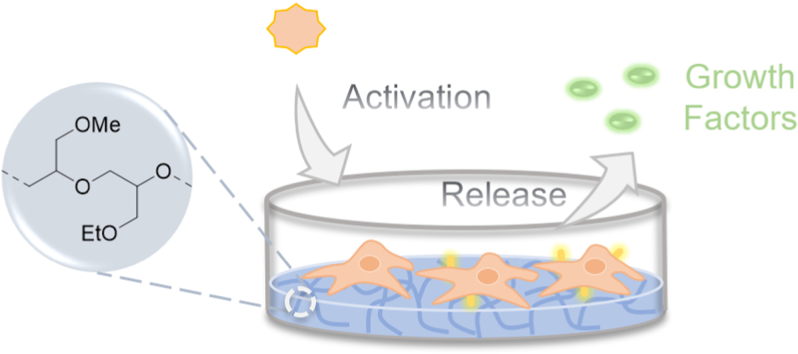

## 1 Introduction

Recent approaches in regenerative medicine are aimed at installing biomaterials with cell-instructive properties to enhance tissue regeneration and repair [1,2]. Immunoengineering is one specific approach targeting functionally active immune cells to support tissue growth and wound repair after injury or disease [[3], [4], [5]]. In that context, biomaterial-instructed immune cells could take over a central part in regenerating injured tissues, such as burned skin or organs with limited regenerative capacities, such as cartilage, cornea, or heart muscle after injury or cardiac infarction [6]. In addition, biomaterial-activated immune cells play a crucial role in tissue remodeling, making them an auspicious tool in regenerative medicine [7]. Besides nanoparticle formulations as vaccine adjuvants, polymeric scaffolds and hydrogels, e.g., from crosslinked dextran and poly(ethylene glycol) (PEG), are progressively engineered as bioactive antigen and costimulatory molecule-releasing biomaterials for immunomodulation [8].

The idea that immune cells, apart from their central function of controlling the inflammatory response against pathogens, exhibit an intrinsic capacity to assist tissue repair has been compellingly demonstrated for the axolotl system. In salamanders, the depletion of macrophages resulted in failure of limb regeneration that could be restored after macrophage replenishment [9]. Specific subtypes of macrophages and dendritic cells (DCs) that could arise under inflammatory conditions and locally restricted to individual organs share monocytes as their common precursor cells and display a similar panel of biomarkers and cytokines [10]. In the absence of antigenic stimulation, specific biomaterials were recently reported to control the immune function of DCs and, thus, direct subsequent tissue regeneration [[11], [12], [13]]. However, only DCs are potent mediators between innate and adaptive immunity due to their ability to educate T cells and, thereby, instructing immunity, anergy, and transplant tolerance [14]. It is further speculated that both macrophages and DCs can prevent extensive fibrosis and dysregulation of extracellular matrix (ECM) components. Hence, tissue reconstruction does not seem to be a primary task of myofibroblasts, which are often associated with scarring, if these cells were either activated by fibroblasts or by other mesenchymal cells such as adipocytes. There are current indications that tissue repair could be rather a function of macrophages and DCs, which also exhibit the capacity to release growth factors to induce fibroblast proliferation and fibroblast-mediated remodeling [15]. Interestingly, specific M2a and M2c macrophages are reported to modulate myofibroblast function [16]. In addition, the ECM microarchitecture shows a certain influence on myofibroblast functions. Whether DCs could also participate in the immunomodulation of myofibroblasts has not yet been clarified. Moreover, there are hints that, both, cytokine and growth factor release are mandatory for tissue repair. For example, Bosurgi et al. [17] showed that the two cytokines, interleukin (IL)-4 and IL-13, released in response to pathogenic helminth infection, require the presence of additional apoptotic cells to induce tissue repair in the lung. Thus, it was concluded that immune cells could shift their programming from pathogen response to tissue repair in the presence of specific signals. Apart from M1 and M2 macrophages, DCs triggered by terminally pathogenic stimuli acquire a pro-inflammatory phenotype of terminally differentiated cells. Yet, the potential of immature DCs to release growth factors and further factors associated with tissue remodeling rather than pathogen defense following exposure to specific surfaces remains to be elucidated.

Apart from the controlled release of bioactive and immunomodulatory molecules through excipient biomaterials, the material itself may also be engineered to exhibit intrinsic bioactivity, e.g., via its geometry/morphology, hydrophobicity, or as a result of specific protein interactions at its surface [5,7]. The exact mechanism of action is far from being fully understood, however, there are receptor candidates that recognize such engineered materials and, intriguingly, models involving immune cells participating in the process of tissue reconstruction. Thus, various biomaterials, such as microspheres, nanofibers, and hydrogels, with different chemistries and architectures, are currently under investigation to promote immune cell-mediated tissue repair [18]. An overall goal *in vivo* is the generation of new tissue resembling the original host tissue instead of fibrous scar formation. Next to macrophages, which have been studied extensively in the context of biomaterial-induced tissue repair [3], DCs are promising candidates because they are also known to release growth and colony-stimulating factors, such as transforming growth factor (TGF)-β1 and granulocyte-macrophage colony-stimulating factor (GM-CSF) which contribute to the construction of ECM and cell differentiation, respectively. Just recently, the central role of *in vivo*-activated human plasmacytoid and myeloid DCs in wound repair and tissue remodeling in the skin, cornea, and heart tissue was discovered [[19], [20], [21], [22]]. Such DCs express upregulated cell surface molecules, e.g., costimulatory cluster of differentiation (CD)40, CD80, CD86, adhesion molecule CD54, and major histocompatibility complex (MHC) class II compound human leukocyte antigen (HLA)-DR. Dendritic cells contribute to all white blood cells at a percentage of 0.2 % and occur at even lower rates in specific tissues such as skin [23]. Due to the limited availability of *in vivo*-activated myeloid DCs, *in vitro-*generated human blood monocyte-derived DCs (moDCs) are commonly used substitutes [24].

Thus, there is a high demand for biomaterials that are able to generate moDCs with *in vivo*-like properties under *in vitro* conditions. Using natural or synthetic polymer-based substrates and coatings for DC culture is a straightforward approach. However, only a few polymer materials have shown high activation potency to yield qualified *in vivo* DC substitutes. In this contribution, we show that poly(glycidyl ether) (PGE) coatings immobilized on conventional culture dishes are efficient materials for the intrinsic *in vitro* activation of human moDCs. In our experimental approach, PGE coatings were able to upregulate costimulatory molecules and enhance the release of growth factors to levels so far only observed by *in vivo* stimulation. As fully synthetic biomaterials, PGE coatings are therefore identified as excellent candidates for moDC activation and, ultimately, biomaterial-induced tissue regeneration.

## 2 Materials and methods

### 2.1 Polymer synthesis

All materials used for polymer synthesis are given in the supplementary material. A detailed procedure for synthesizing block copolymers **B1** and **B2** can be found in our previous report [25]. In brief, block copolymers were synthesized via the monomer-activated anionic ring-opening polymerization of the randomly copolymerizing [26] glycidyl methyl ether (GME) and ethyl glycidyl ether (EGE) followed by the sequential addition of the photo-reactive comonomer 4-(2,3-epoxypropoxy) benzophenone (EBP). Thus, poly(GME-*ran.*-EGE)-*block*-poly(EBP) block copolymers with GME:EGE comonomer ratios of 1:1 (**B1**) and 1:3 (**B2**), which were used to fabricate PGE brush coatings were obtained (Fig. S1a). The synthesis and characterization of statistical terpolymers **G1**, **G2**, and **G3** are described in detail in the supplementary material and were conducted according to our previously reported protocols with slight modification [25]. Initially, allyl-functional poly(GME-*stat.*-EGE-*stat.*-AGE) terpolymers were obtained via the statistical copolymerization of GME, EGE, and allyl glycidyl ether (AGE). The obtained terpolymers were subsequently post-functionalized with amine groups via UV-induced radical thiol-ene coupling of 2-aminoethanthiol hydrochloride to AGE allyl groups. In the next step, the amine-functional terpolymers were equipped with photo-reactive BP units via amide coupling using the carboxy-functional benzophenone derivative 4-benzoylbenzoic acid (4-CBP). Thus, poly(GME-*stat.*-EGE-*stat.*-AC-BP) terpolymers with GME:EGE comonomer ratios of 1:1 (**G1**), 1:3 (**G2**), and 1:7 (**G3**), which were used to fabricate PGE gel coatings, were obtained (Fig. S1b). Random poly(GME-*ran.*-EGE) copolymers with GME:EGE comonomers ratios of 1:1 (**S1**) and 3:1 (**S2**) were synthesized in analogy to PGE block copolymers **B1** and **B2** by omitting the sequential addition of EBP according to our established protocols (Fig. S1c) [25,26].

### 2.2 Surface preparation and characterization

A detailed description of the materials and methods used for surface preparation and characterization is given in the supplementary material. For cell culture experiments on polymer-modified substrates, conventional 35 mm diameter Falcon® PS culture dishes - so-called suspension dishes - from Th. Geyer GmbH & Co KG (Berlin, Germany) were coated with PGEs either via the adsorption/immobilization grafting-to approach, in the case of PGE brushes, or via spin coating and UV-induced immobilization/crosslinking, in the case of PGE gels. To allow detailed structural characterization via spectroscopic ellipsometry (SE), water contact angle (CA), and atomic force microscopy (AFM), all PGE coatings were additionally prepared in an analogous fashion on PS-coated silicon (Si) wafer model substrates. Si wafers were therefore spin-coated with thin PS layers (∼50 nm) from 1 % (w/w) solution of the Falcon® PS culture dish material in toluene. PGE brushes on PS dishes (Falcon® PS dishes) or PS-coated Si-model substrates were fabricated via the adsorption/immobilization grafting-to approach [25,27]. In brief, PS substrates were incubated in dilute block copolymer solutions (67.5 μg mL^−1^) prepared in aqueous EtOH (32 % (v/v) EtOH for **B1** and 46 % (v/v) EtOH for **B2**) for 1 h. The polymer solutions were subsequently discarded, and the surfaces briefly immersed in water to remove excess polymer solution. After drying under a stream of N_2_, the substrates were irradiated with UV light (365 nm) for 160 s to covalently immobilize the physically adsorbed PGE brush layers via their photo-reactive EBP anchor blocks. The surfaces were then washed with EtOH to extract non-immobilized PGE chains until the thickness of the coatings was constant (∼1 d). PGE gels on PS dishes (Falcon® PS dishes) or PS-coated Si-model substrates were fabricated via spin coating of statistical terpolymers **G1**-**G3** from EtOH solution (1 % (w/w)). Thinner **G1a** gels were prepared accordingly by spin coating from a more dilute EtOH solution (0.1 % (w/w)). The substrates were subsequently irradiated with UV light (365 nm) for 160 s to immobilize and simultaneously crosslink the PGE coatings. To remove non-immobilized PGE chains, the substrates were extracted with EtOH until the dry thickness of the gels was constant (∼5 d). On PS-coated Si wafer model substrates, the dry thickness of the PGE coatings was measured by SE before and after UV irradiation as well as during extraction with EtOH. The wettability of the obtained coatings was characterized by static water contact angle (CA) measurements under ambient conditions at 20 °C. AFM measurements were performed in a liquid chamber at 37 °C. Analogously prepared PGE-coated PS dishes were used for moDC culture experiments.

### 2.3 Ethical approval

For experiments with human blood samples and blood-derived cells, approval by the ethics committee of the Charité – University Medicine Berlin was obtained (no. EA1/201/09). Anonymized blood samples were obtained from the German Red Cross blood donation service Berlin with informed written consent from all participants. All studies were in accordance with the Helsinki guidelines. No part of these studies was conducted outside of Germany.

### 2.4 Preparation of blood-derived monocytes and generation of moDCs

Human PBMCs were obtained by Ficoll gradient (PAA Laboratories) centrifugation from buffy coats of healthy donors provided by the German Red Cross blood donation service, Berlin. By depletion of contaminating cells, monocytes were magnetically isolated from PBMC (monocyte isolation kit II, Miltenyi Biotec) and then differentiated into immature moDCs during culture on tissue culture polystyrene dishes in RPMI 1640 media supplemented with 2 mM l-glutamine, 100 IU/ml penicillin, 100 μg/ml streptomycin (all PAN Biotech) and 10 % (v/v) heat-inactivated FCS (Biochrom) for 6 days. For effective moDC differentiation, 100 ng/ml GM-CSF and 10 ng/ml IL-4 [28] (both Miltenyi Biotec) were added to the medium and replenished every 2 days according to a standard protocol for experimental and clinical studies using human DCs [24]. These moDCs are a distinct subtype of DCs, with some overlapping functions, but also important different functions than classical (conventional) DC subsets as discussed by Collin and Bigley [29].

### 2.5 MoDC activation on biomaterial surfaces

Control experiments with uncoated cell culture substrates were performed on conventional 35 mm Nunclon™ Delta (Nunc) or Corning® (Corn) TCPS dishes supplied by ThermoFisher Scientific and Sigma Aldrich, respectively. Before seeding, PGE-coated culture dishes were disinfected with aqueous EtOH (70 % EtOH) for 15 min under the laminar flow bench. The dishes were subsequently washed with sterile phosphate-buffered saline (PBS) three times, closed, and stored in the safety cabinet with the remaining volume of PBS to keep the coatings hydrated for up to two days before culture experiments were conducted. Immature moDCs were seeded on PGE brush- and gel-coated substrates and TCPS controls at a seeding density of 5.7x 10^5^ cells cm^−2^ in RPMI 1640 media supplemented with 2 mM l-glutamine, 100 IU/ml penicillin, 100 μg/ml streptomycin (all PAN Biotech) and 10 % (v/v) heat-inactivated FCS (Biochrom). 100 ng/ml Lipopolysaccharide (LPS) from *E. coli* (O26:B6, Sigma-Aldrich) was used as positive control in some experiments. Media was replenished every 2 days. After 4 days, half of the media was collected for direct analyses of MMP-1, -7, and -9, EGF, and TGF-β1 by ELISA. On day 6, cells were harvested by washing with cold PBS, counted, and prepared for direct flow cytometry analysis.

### 2.6 Flow cytometry

All monoclonal antibodies were obtained from BD Bioscience and used at concentrations recommended by the manufacturer. Dead cells were excluded from analysis by staining with 7-AAD (BD Bioscience). 2x 10^5^ cells/vial were incubated with Fc Block (BD Bioscience) and mouse anti-human CD86 BB515 (2331), mouse anti-human CD40 APC (5C3), mouse anti-human HLA-DR PerCPCy5 (G46-6), mouse anti-human CD14 PE (M5E2), mouse anti-human CD1c FITC (BDCA1), mouse anti-human CD83 APC (HB15), mouse anti-human CD206 FITC (19.2), mouse anti-human CD367 AF647 (I3-612), mouse anti-human CD1c FITC (BDCA1), mouse anti-human CD83 APC (HB15), mouse anti-human CD206 FITC (19.2), mouse anti-human CD367 AF647 (I3-612), mouse anti-human PD-L1 FITC MIH37 and mouse anti-human PD-L2 PE (MIH1) or appropriate isotype controls for mIgG1 BB515, mIgG1 APC, mIgG2a PerCPCy5 and mIgG2a PE for 30 min on ice. For viability analyses, cells were incubated in the presence of annexin V FITC and 7-AAD in 2.5 mmol Ca^2+^ containing PBS. After washing, cells were analyzed with a FACS Accuri C6 flow cytometer (BD Bioscience) using the BD Accuri C6 software.

### 2.7 ELISA

Supernatants harvested on day 4 of moDC culture on PGE-coated surfaces and TCPS controls (Nunc and/or Corn) were analyzed for MMP-1, -7 and -9, TGF-β1, and EGF (DuoSet, R&D Systems) by sandwich ELISA. Values for OD were detected with a microplate reader Infinite (Tecan Group) at 450 nm and a reference wavelength of 540 nm. Concentrations in pg mL^−1^ were calculated from a 7-point calibration curve for each analyte.

### 2.8 Statistical analysis

Water CAs of PGE brushes **B1** and **B2** were statistically compared using the non-parametric Mann-Whitney-U test (Fig. 2b). Statistical comparison between PGE gels **G1a**, **G1b**, **G2**, and **G3** was performed using the non-parametric Kruskal-Wallis test with a subsequent Dunn-Bonferroni post hoc test (Fig. 2b). Mean fluorescence intensities and fold changes determined by flow cytometry (Fig. 5, Fig. **6** and Fig. **S10**) as well as growth factor and MMP release determined by ELISA (Fig. 7, Fig. **8** and Fig. **S11**) were statistically compared to Nunc TCPS controls using the non-parametric Mann-Whitney-U test. In each statistical comparison values of *p* < 0.05 were considered significant (*, *p* < 0.05; **, *p* < 0.01; ***, *p* < 0.005).

## 3 Results and discussion

### 3.1 Synthesis of poly(glycidyl ether)s as materials for DC activation

**Fig. 1:**
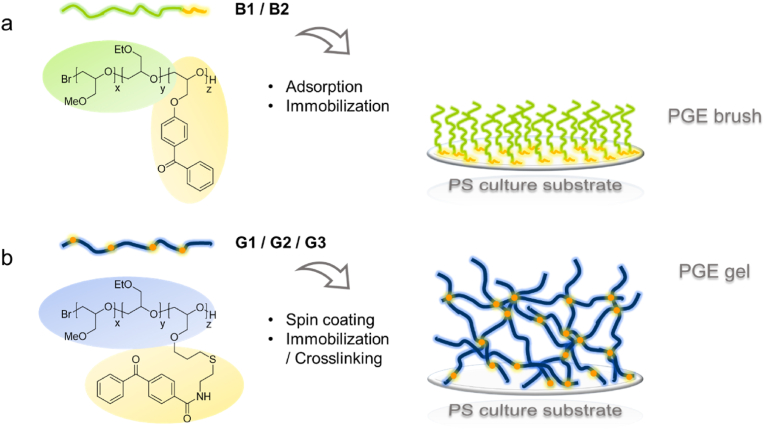
(a) Chemical structure of poly(GME-*ran.*-EGE)-*block*-poly(EBP) block copolymers **B1-2** and schematic design of PGE brush coatings on PS culture substrates after the adsorption/immobilization grafting-to method [25,27,45]. (b) Chemical structure of statistical poly(GME-*stat.*-EGE-*stat.*-AC-BP) **G1-3** terpolymers and schematic design of PGE gels on PS culture substrates after spin coating and immobilization/crosslinking.

Reports on the intrinsic *in vitro* activation and maturation of DCs through natural or synthetic biomaterials are rather scarce. Most notably, chitosan-, agarose- and poly(lactic-co-glycolic acid) (PLGA)-based culture substrates have shown to promote the differentiation and activation of DCs [[30], [31], [32], [33], [34], [35], [36], [37]], whereas most conventional cell culture materials, such as tissue culture-treated polystyrene (TCPS) and non-treated PS, polycarbonate (PC), poly(tetrafluoro ethylene) or poly(dimethyl siloxane), do not significantly enhance DC activation without additional bacterial liposaccharide (LPS) treatment [[38], [39], [40]]. As fully synthetic polymer materials, poly(glycidyl ether) (PGE) coatings are non-toxic, biocompatible and have already demonstrated to facilitate the adhesion and proliferation of adherent mammalian cells, including human primary cells [[41], [42], [43], [44]]. In the latter context, the immunomodulatory effect of PGE coatings on various cell types, such as moDCs, is an important parameter with regards to their use as materials in tissue engineering in general. To study their potential for *in vitro* moDC activation, we prepared PGE brush and unprecedented gel coatings on conventional PS suspension culture substrates via “grafting-to” approaches. For this, PGE copolymers were synthesized in a controlled manner via the monomer-activated anionic ring-opening polymerization of the two randomly copolymerizing monomers glycidyl methyl ether (GME) and ethyl glycidyl ether (EGE) [26]. To obtain PGEs with different hydrophilicity and, thus, afford PGE coatings with varied physicochemical properties, we adjusted the comonomer ratio of the more hydrophilic GME compared to the EGE monomer. As illustrated in Fig. 1, two types of terpolymers comprising photo-reactive benzophenone (BP) moieties, which are used to covalently immobilize or simultaneously crosslink PGEs on PS culture substrates via simple UV light irradiation, were obtained applying two different strategies. In the first approach, PGE block copolymers bearing a short BP anchor block were synthesized via the sequential anionic living polymerization of a mixture of GME and EGE, and then the photo-reactive comonomer 4-(2,3-epoxypropoxy) benzophenone (EBP) (Fig. S1a) [25]. Such block copolymers comprising a hydrophobic EBP anchor block can be used to self-assemble and covalently immobilize PGE brushes (Fig. 1a) on hydrophobic polymeric materials, e.g., PS and PC culture substrates [25,45]. Whereas the direct anionic copolymerization of BP-based monomers is feasible in a sequential manner, statistical copolymerization of EBP, which would be necessary to obtain crosslinkable PGE terpolymers for the fabrication of gel coatings, is not possible due to the limited stability of BP moieties under the applied polymerization conditions [46]. Therefore, in the second approach, statistical terpolymers bearing BP moieties along the PGE backbone were synthesized via the two-step post-functionalization of allyl-bearing terpolymers composed of GME, EGE, and allyl glycidyl ether (AGE) according to a modified protocol based on our previous report (Fig. S1b) [25]. Such terpolymers can be used to immobilize and simultaneously crosslink PGE gels (Fig. 1b) to plastic culture substrates, e.g., via spin coating and subsequent irradiation with UV light.

**Fig. 2:**
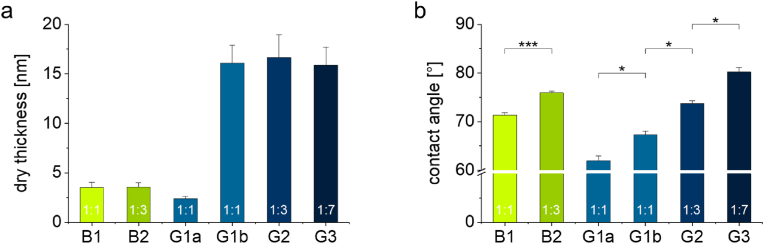
(a) Dry layer thickness of PGE brush (**B1** and **B2**) and gel coatings (**G1a**, **G1b**, **G2**, and **G3**) determined by spectroscopic ellipsometry (SE) on PS-coated silicon wafer model substrates which were identically coated as PS-based cell culture ware (n = 6, error bars indicate standard deviation (SD)). (b) Wettability of PGE brush (**B1** and **B2**) and gel coatings (**G1a**, **G1b**, **G2**, and **G3**) determined by static water contact angle (CA) measurements on PS-coated silicon wafer model substrates (n = 6, error bars indicate SD). CA differences between different brush or gel coatings were analyzed for statistical significance with the non-parametric Kruskal-Wallis with subsequent post-hoc test, respectively. Values of *p* < 0.05 were considered significant (*: *p* < 0.05, ***: *p* < 0.005). (For interpretation of the references to color in this figure legend, the reader is referred to the Web version of this article.)

The composition and molecular weight data of all synthesized PGE terpolymers are summarized in Table S1 and close to the target values. For the fabrication of brush coatings, according to our previous reports, block copolymers (Fig. 1a) of around 30 kDa with GME:EGE comonomer ratios of 1:1 (**B1**) and 1:3 (**B2**), respectively, and an average of 5 EBP repeating units were targeted (Table S1, entry 1–2) [25]. Statistical PGE terpolymers (Fig. 1b) of around 40 kDa with GME:EGE comonomer ratios of 1:1 (**G1**), 1:3 (**G2**), and 1:7 (**G3**), respectively, and an average of 1.5 mol-% AGE repeating units were targeted to fabricate a new type of PGE gel coatings (Table S1, entry 3–5). In addition to photo-reactive block copolymers and terpolymers, BP-free copolymers (Fig. S1c, Table S1) with GME:EGE ratios of 1:1 (**S1**) and 3:1 (**S2**) were synthesized to serve as soluble PGE controls for moDC activation.

### 3.2 Functionalization of polystyrene culture substrates

Two different protocols were utilized to immobilize PGE coatings on PS culture substrates. PGE brushes were fabricated via an established adsorption/immobilization grafting-to approach of PGE block copolymers **B1** and **B2** onto PS substrates from dilute aqueous EtOH solution [25,27,45]. In brief, PS substrates were incubated in the respective PGE solutions for 1 h, washed with water, and irradiated with UV light at 365 nm in the dry state for 160 s. Thereby, the self-assembled brushes are covalently immobilized via C,H-insertion of the photo-reactive EBP anchor block into the polymer backbone of the PS surfaces. After extraction of unbound polymer chains with EtOH overnight, stable PGE brush layers in the low nanometer range were obtained. A new type of PGE coating architecture was obtained with nanometer-thin gels by spin coating statistical PGE terpolymers **G1-G3** onto PS substrates from EtOH solution at a polymer concentration of 1 % (w/w). The layers were immobilized with simultaneous crosslinking via UV irradiation for 160 s and subsequently extracted with EtOH for 5 days until the thickness of the coatings adopted steady values. The thickness and wettability of the coatings, which were determined on additionally prepared PS-coated silicon wafer model substrates using spectroscopic ellipsometry (SE) and static water contact angle (CA) measurements, respectively, are summarized in Fig. 2.

As shown in Fig. 2a, PGE brush coatings **B1** and **B2** with an average dry thickness of ∼3.5 nm were obtained. Under aqueous conditions, the grafting density of the hydrated PGE chains (*M*_n_ = 30 kDa) is high enough (∼0.09 chains nm^−2^) for the polymers to adopt a brush-like conformation and to fully cover the PS substrate surface [25,27,42,45]. Thus, potential activating effects of the underlying PS culture substrate on moDCs can be excluded. Furthermore, based on the literature, DC activation levels on hydrophobic PS culture substrates are in the same range as TCPS control substrates [[38], [39], [40]]. With an average of ∼15 nm, the dry thickness values of PGE gels **G1b**, **G2**, and **G3** were markedly higher than those of PGE brushes (Fig. 2a). In addition to thick (∼15 nm) **G1b** gels, thin (∼2.5 nm) **G1a** gels were prepared with the same terpolymer **G1** from a more dilute solution (0.1 % (w/w)) in EtOH by spin coating. These **G1a** coatings were prepared to improve cell adhesion during moDC activation. As illustrated in Fig. 1b, static water CAs range from about 60 to 80° and reflect the different hydrophilicities of the coatings. As evident from statistical analysis, differences in CAs are significant among brushes and gels, respectively, demonstrating that the coatings' wettability can be efficiently adjusted via the copolymers’ GME:EGE comonomer ratios. Notably, the wettability of PGE coatings with comparable comonomer compositions is slightly higher for PGE gels compared to brushes, which is reflected in the lower water CAs of the gels. This invariable difference can be attributed to the distinct polymer chain conformation within disparate brush and gel coating architectures (Fig. 1) which can exhibit vastly different degrees of PGE chain hydration. Interestingly, thin **G1a** gels exhibit lower water CAs than thicker **G1b** gels (Fig. 2b). Such an increase in wettability is likely due to the decreased degree of crosslinking within the thin **G1a** gels and the hence associated higher degree of hydration of the less constrained PGE chains. Nevertheless, CAs of PGE brushes and gels are in the same realm and located in the range of common tissue culture substrates.

**Fig. 3:**
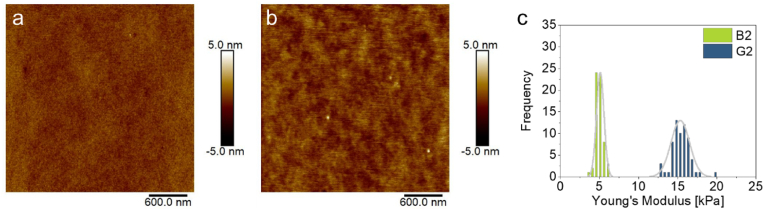
Representative morphological images of PGE brush **B2** (a) and gel **G2** (b) coatings on PS-coated silicon wafer model substrates and corresponding Young's Moduli (c) measured in water at 37 °C via AFM. (For interpretation of the references to color in this figure legend, the reader is referred to the Web version of this article.)

To determine the morphology and mechanical properties of the coated substrates, PGE coatings were characterized by atomic force microscopy (AFM). Representative topological images of a brush (**B2**) and a gel (**G2**) coating, both with a GME:EGE comonomer ratio of 1:3 (Table S1), on PS-coated silicon wafer model substrates in water at 37 °C, simulating cell culture conditions, are illustrated in Fig. 3. Whereas **B2** brushes exhibit a rather homogeneous and smooth morphology (Fig. 3a, Fig. S2), **G2** gels present slightly rougher and laterally more inhomogeneous surfaces (Fig. 3b, Fig. S2). Because both coatings are composed of PGEs with similar composition but different architecture, the observed discrepancy can be attributed to the less defined nature of the randomly crosslinked and immobilized gels as compared to the more controlled nature of the immobilization method used for self-assembly of PGE brushes. Generally, gels exhibit higher degrees of swelling than the corresponding brush coatings. It is well known from previous studies that the morphology of PGE coatings on Si wafers and PS dishes are highly comparable, except for the intrinsically enhanced surface roughness of PS culture dishes compared to spin-coated PS on Si wafers [45,47].

Quantitative nanomechanical mapping (QNM) with a pyramidal AFM tip revealed marked differences in deformation, elasticity, and adhesion when comparing the brush with gel coatings (Fig. S3). Interestingly, the morphology and mechanical properties were comparable among the investigated brush and gel coatings, respectively (Figs. S4–S7), which indicates that the coating properties are mainly governed by the coating architecture rather than the GME:EGE comonomer composition. Since the mechanical properties of thin coatings with nm-scale thicknesses can be markedly influenced by the underlying substrate when measurements are conducted with a pyramidal nm-sized AFM tip (*r* ∼ 10 nm), we further conducted nanoindentation measurements using a spherical colloidal SiO_2_ probe (*d* ∼ 4.8 μm). Representative Young's moduli of **B2** brush and **G2** gel coatings are illustrated in Fig. 3c and the supplementary material (Fig. S8). In contrast to QNM, nanoindentation measurements provide more realistic absolute elasticity values in the kPa range due to the larger contact area between the colloidal probe and the coatings, thus, largely excluding the PS substrate bias evoked by the pyramidal tip. Consequently, surface-tethered and crosslinked PGE gels exhibit up to three times higher Young's moduli than purely end-tethered PGE brushes in the low kPa-range (Fig. 3c, Fig. S8), which is grounded in the crosslinked, more rigid gel architecture. The mechanical properties, i.e., Young's Moduli, of our PGE coatings are well within the range of natural soft tissues and a realistic approximation of the mechanical cue experienced by immune cells cultured on the coated substrates. In the following, we examine the potential of the PGE coatings for the activation of moDCs to discern their applicability for immunoengineering and to draw structure-property relations between the physical PGE coating properties, e.g., hydrophilicity and elasticity, and their moDC activation potential.

### 3.3 Activation of dendritic cells by PGE coatings

**Fig. 4:**
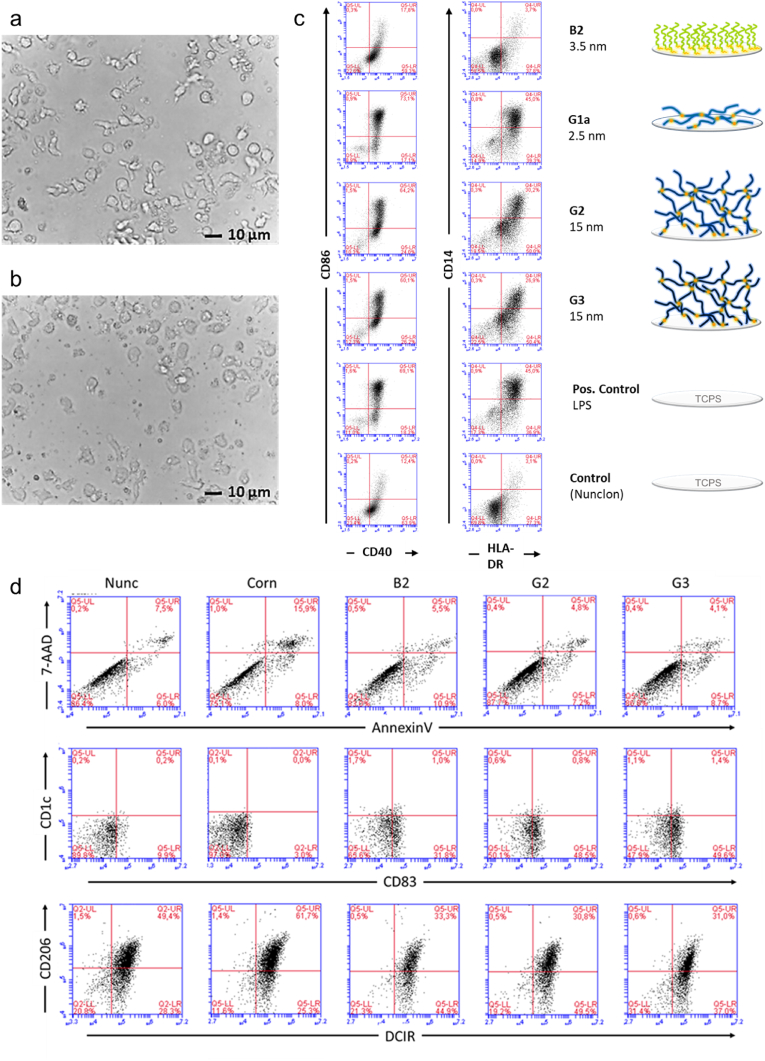
Activation of immature moDCs cultured on **B2** brush and **G1a**, **G2**, and **G3** gel coatings determined by morphological analysis from bright field microscopy images and flow cytometry (n = 5 donors). Representative brightfield microscopic images of moDCs after 4 days of culture on **G2** gel (a) and Nunc TCPS (b) substrates. (c) Flow cytometry dot plot analyses of the moDC activation status from one representative healthy donor by means of upregulation of markers CD86, CD40, CD14, and HLA-DR on day 6 of culture with a schematic illustration of the respective surface coatings next to it. (d) Flow cytometry analyses of moDC viability and phenotype after culture on **B2** brush and **G2** and **G3** gel coatings; shown are dot plots from one representative healthy donor (n = 3 donors).

MoDCs were generated *in vitro* from human peripheral blood monocytes (PBMCs) by IL-4 and GM-CSF treatment, as reported previously [24,48]. These moDCs were then exposed to PGE brush and gel coatings immobilized on conventional PS culture dishes. Whereas cells adhered well on PGE coatings with GME:EGE comonomer ratios of 1:3–1:7, adhesion was insufficient and strongly hampered on more hydrophilic **G1b** gel and **B1** brush coatings (GME:EGE ∼ 1:1), respectively. However, decreasing the thickness of the gel from ∼15 to only ∼2.5 nm drastically improved cell adhesion and afforded the culture of moDCs on substrates with thin **G1a** coatings. Even though **G1a** gels exhibit enhanced surface wettability compared to **G1b** gels (Fig. 2b), the latter presumably experience more bulk hydration than the former due to the stronger dehydrating effect of the hydrophobic PS substrate on the thinner **G1a** layers. Despite lower water CAs, which, in this case, reflect wettability rather than hydrophilicity, **G1a** gels are overall less hydrated/hydrophilic than **G1b** gels and therefore exhibit improved cell-adhesive properties. Interestingly, cells acquired the characteristic *in vivo*-like morphology of moDCs, particularly when cultured on PGE gel coatings as compared to conventional Nunclon™ Delta (Nunc) TCPS control dishes. At the end of cytokine-mediated moDC-differentiation, the majority of cells is veiled with typical dendritic cell morphology. Even after 4 days, a substantial number of moDCs displayed veils and moDC-characteristic cytoplasm protrusions, as representatively shown in Fig. 4a on **G2** gel coatings compared to the Nunc TCPS control (Fig. 4b). This is attributed to the high potential of primary monocytes from human blood to differentiate and mature into functional, competent moDCs compared to cell lines such as THP-1, MUTZ-3 or U937 which are pre-monocytic and leukemic in origin. The degree of activation in moDCs is known to be mirrored by the expression level of cell surface markers associated with antigen presentation and co-stimulation. Thus, in the first screening experiment, human MHC–II–associated molecule HLA-DR, costimulatory molecules CD86 and CD40, and the monocyte marker CD14 were accessed via flow cytometry on day 6 of moDC culture on various substrates (Fig. 4c). Compared to moDCs harvested from commercially available Nunc TCPS dishes, moDCs harvested from brush and gel surfaces exhibited enhanced cell surface marker expression. As illustrated for one representative healthy donor in Table 1, both parameters, mean fluorescence intensity (MFI) and % positive cells, were upregulated after exposure to PGE brush as well as gel coatings, with the most substantial effect shown in response to thin **G1a** gels. On the latter surface, the range of upregulation in MFI compared to the Nunc control was higher for CD86 (>10-fold change) than for CD40 (∼2-fold change) and HLA-DR (∼3-fold change). When analyzing % positive cells, fold change values for CD86, CD40, and HLA-DR were less pronounced than fold change values by MFI but showed a similar tend. This may reflect the observation that these receptors were amplified on the cell surface by exposure to gel and brushes coatings rather than cells originally deficient for CD86, CD40 and HLA-DR starting to express these molecules *de novo*.

**Fig. 5:**
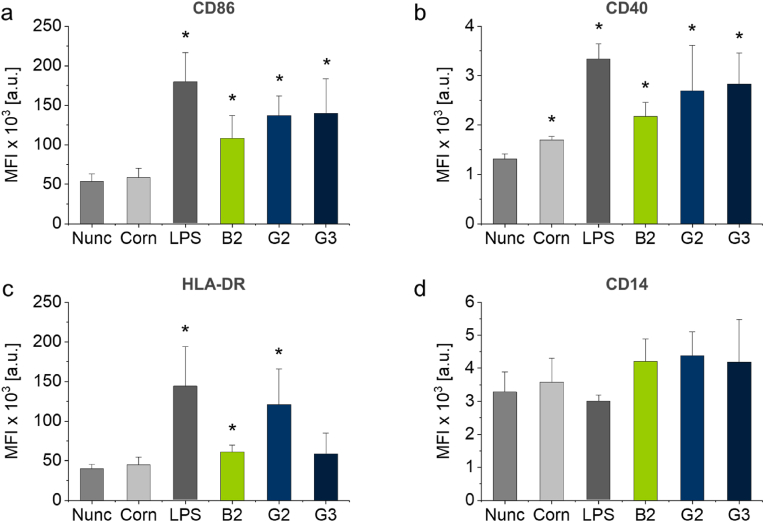
Mean fluorescence intensity (MFI) of geometric mean for costimulatory molecules CD86 (a), CD40 (b) and HLA-DR (c) and monocyte marker CD14 (d) of moDCs cultured on **B2** brush as well as **G2** and **G3** gel coatings and on TCPS control substrates (Nunc, Corning® (Corn)) as well as of LPS-activated moDCs (positive control) determined by flow cytometry. Columns represent the mean of results from n = 4 healthy donors and are plotted together with their SD (error bars). Statistical significance was tested via the non-parametric Mann-Whitney-U test against the Nunc TCPS control. Values of *p* < 0.05 were considered significant (*: *p* < 0.05).

**Table 1:** Representative values for mean fluorescence intensity (MFI) and % positive cells (% pos.) of costimulatory molecules CD86, CD40, and HLA-DR, as well as monocyte marker CD14 of moDCs cultured on **B2**, **G1a**, **G2**, **G3**, and a Nunc TCPS control taken from one healthy donor of the screening experiment.

Substrate	CD86		CD40		HLA-DR		CD14	
	MFI	% pos.	MFI	% pos.	MFI	% pos.	MFI	% pos.
B2	38,655	18	7205	77	23,271	42	1702	4
G1a	304,785	74	12,652	90	56,779	84	11,585	46
G2	170,536	62	12,412	86	54,071	77	7230	27
G3	235,452	66	14,358	88	60,833	80	9866	31
Nunc	29,758	13	6244	76	18,291	30	2429	3

Since cells resembled a more monocytic phenotype when cultured on thin **G1a** gels, as evidenced by enhanced CD14 levels (Table 1), these substrates were excluded from the next series of experiments. MoDCs harvested from the remaining brush and gel surfaces were further analyzed for viability (Fig. 4d). As shown in dot plots of Annexin V and 7-AAD costainings, brush and gel coatings enhanced cell viability as both - percentage of dead cells (double positives) and apoptotic cells (single annexin V positives) decreased as compared to uncoated dishes (Corning). Since the gel but not the brush coatings increased CD14 (Fig. 4c), the specific phenotype of the moDCs after culture was further investigated by staining of moDC lineage markers. In human blood, CD1c identifies conventional DC2 and DC3 but is not expressed on cDC1 and monocytes [49]. After exposure to **B2**, **G2** and **G3** dishes, only a few cells expressed CD1c. Interestingly, the classical marker of moDC maturation, CD83, was found elevated by brush and gel coatings (Fig. 4d). DC immunoreceptor (DCIR) is not only expressed on immature and mature MoDCs but also on monocytes and macrophages. Recently it was shown that DCIR is internalized efficiently into human moDC after activation [50]. In our experiments DCIR decreased slightly from the cell surface after exposure to **B2**, **G2** and **G3** dishes. Even more pronounced, macrophage mannose receptor type C (CD206) decreased by exposure to gel and brush coatings, indicating a less phagocytic phenotype.

Although the PGE-modified culture substrates were fabricated from sterile-filtered aqueous EtOH solutions under laboratory conditions and, therefore, potential bacterial contaminations are unlikely, we tested the endotoxin levels of the substrates to exclude any non-material-related moDC activation through abundant LPS. As shown in Fig. S9, endotoxin levels detected on **G2-**and **G3-**functionalized culture dishes with varying gel thickness only showed negligible endotoxin levels (∼0.1 EU mL^−1^) at the lower end of the detection limit of the assay and similar to non-functionalized, commercial control dishes approved for cell culture. The Food and Drug Administration (FDA) limit for medical devices that directly or indirectly contact the cardiovascular and lymphatic system is set at 0.5 EU mL^−1^ [51]. Therefore, contamination during coating fabrication with moDC-activating bacterial LPS can be excluded. In the next series of flow cytometry experiments, sampling analyses of different human donors (n = 4) plotted as MFI revealed that PGE gels increase moDC-specific markers significantly and more drastically than PGE brushes and TCPS control dishes (Fig. 5). As a result, the antigen density of CD86, CD40, and HLA-DR on moDCs was strongly enhanced by **G2** and **G3** coatings. CD14 was just slightly modified after adhesion to PGE substrates, which indicates the presence of a stable phenotype. In the literature, high levels of costimulatory molecules, such as CD86 and class II molecules, were also reported for human blood monocyte-derived DCs treated with different biomaterial films of chitosan or poly(DL-lactic-co-glycolic acid) (PLGA, molar ratio = 75:25), however, only in the context of DC maturation and biocompatibility [33]. Along these lines, addressing the cytocompatibility of hydrogels for a 3D immune-competent cell culture model to mimic human subcutaneous tissue, geltrex® matrix - a reduced growth factor basement membrane extract - and 0.25 % (w/v) agarose were found to be appropriate materials using immature MUTZ-3 DCs [52]. The observed moDC activation levels upon exposure to PGE surfaces were higher than in any biomaterial surface experiment reported previously. In a study by Park and Babensee [33], fold change for CD86 on moDCs treated by different biomaterial films was in the range of 2 or less. In mice, unstimulated cells of the immature dendritic cell line JAWSII cultured on functionalized hydrogels demonstrated less than 40 % [53] compared to 66 % positive cells for CD86 in our experimental approach using **G3** gels (Table 1). In general, the stimulation level of CD86, CD40, and HLA-DR observed in this series of experiments is comparable to the effect of pathological stimuli such as ligands for Toll-like receptors 4 and 2, LPS, and peptidoglycans on generated/matured DCs [48].

**Fig. 6:**
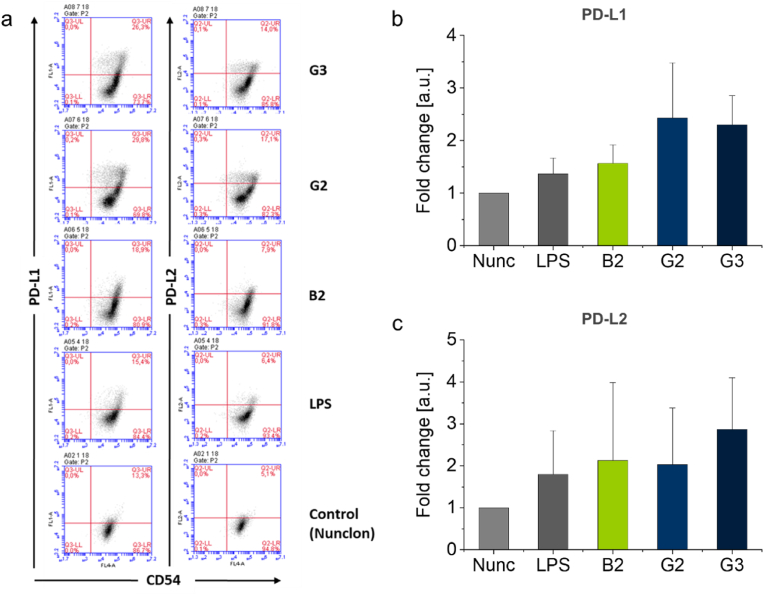
Expression of immune-control associated molecules on CD54 positive moDCs cultured on **B2** brush as well as **G2** and **G3** gel coatings determined and analyzed by flow cytometry from n = 3 healthy donors on day 6 of culture. Flow cytometry dot plot analysis of PD-L1 and PD-L2 from one representative healthy donor (a) and fold change analysis of PD-L1 (b) and PD-L2 (c) expression (geometric mean) from a mean of 3 donors compared to Nunc TCPS control substrates and LPS-activated moDCs (positive control).

The immune-inhibitory molecules programmed cell death (PD)-L1 and PD-L2 on DCs are reported to interact with their checkpoint receptors on T cells and thereby participate in T cell mediated transplant tolerance [54]. Furthermore, PD-L1 involvement was shown to control skin DC activation and subsequent T cell activation [55,56]. Thereby DCs deliver negative feedback mechanisms upon DC activation to prevent an exaggerated immune activation after stimulation. Here, we investigated expression of both molecules after 6 days of moDC exposure to brushes and gels. To verify that expression of PD-L1 and PD-L2 might not be due to contaminating cells within the moDC culture, the cells were co-stained and identified by CD54, a marker well known to be expressed on human moDCs [23]. The level of expression for PD-L1 and -L2 on moDCs was moderately increased by **B2**, **G2**, and **G3** coatings (Fig. 6). In contrast, soluble PGEs **S1** and **S2** (Fig. S1c, Table S1) at a concentration of 10 μg mL^−1^, which corresponds to the amount of PGE immobilized in gel coatings, did not show any effect on the investigated immune-inhibitory molecules, independent of the PGE comonomer ratio and hydrophilicity (Fig. S10). This indicates that moDCs are not intrinsically activated by the PGE structure alone but that the mechanical cue exerted on moDCs by immobilized PGE coatings with distinct polymer chain orientation is essential for moDC activation [57].

**Fig. 7:**
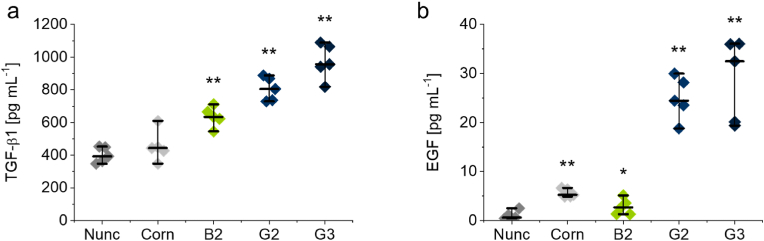
Release of transforming growth factor (TGF)-β1 (a) and epidermal growth factor (EGF) (b) by moDCs cultured for 4 days on **B2**, **G2**, **G3**-coated culture dishes and TCPS controls (Nunc, Corn). Protein levels were analyzed by ELISA in supernatants of moDCs generated from primary PBMCs of n = 5 healthy donors. Differences of TGF-β1 and EGF values were analyzed for statistical significance and compared to Nunc TCPS controls using the non-parametric Mann-Whitney-U test. Values of *p* < 0.05 were considered significant (*: *p* < 0.05, **: *p* < 0.01).

Parallel expression of co-stimulatory and co-inhibitory molecules on moDCs was observed by different authors [55,56] and seems to represent activation and induction of shutdown of potentially harmful immune reactions in the human body shortly after inflammatory events. However, studies on the material-induced expression of PD-L1 and -L2 have not been reported in the literature so far.

Activation of moDCs under *in vivo* conditions is a process only achieved by exposure to pathogenic organisms such as bacteria, protozoa, viruses, or fungi and by exposure to specific xenobiotica. Numerous publications exist in the literature on the secretion of cytokines, chemokines, and growth factors from moDCs after exposure to total pathogens, pathogen-associated molecular patterns (PAMP), as well as chemicals [10,58].

Since only little is known regarding the release of growth factors by DCs in contact with polymeric biomaterials, we analyzed these soluble factors released by surface-activated moDCs in the next series of experiments. Simultaneous to activation marker expression, as determined by flow cytometry, moDCs released transforming growth factor (TGF)-β1 and epidermal growth factor (EGF). For TGF-β1 and EGF, the amounts found in the supernatant of moDCs cultured on PGE substrates for 4 days were significantly enhanced compared to TCPS control dishes, and more evidently for PGE gel rather than brush coatings (Fig. 7). Induction of growth factors in DCs by polymer materials *in vitro* has previously not been reported, but the role of growth factors was addressed in a study using mouse DCs. In this report [53], PEG hydrogels were loaded with external TGF-β1 to reduce the immune response to material carriers. The important role of DC-derived TGF-β1 in tissue regeneration *in vivo* was demonstrated in a study using diphtheria toxin receptor transgenic mice. These animals were devoid of DCs and showed delayed wound closure associated with decreased wound levels of TGF-β1 [22].

With respect to EGF, emerging data indicate an important role for this growth factor in tissue repair as well. For immune cells activated by tissue damage, such as acute lung injury during worm infections, involvement of EGF-like molecules in homeostasis and regeneration was suggested [59]. A related growth factor, vascular endothelial growth factor (VEGF), is secreted by THP-1-derived macrophages if stimulated by 3D nanofibrous PS scaffolds (NPSs) [60]. In addition to growth factors, pro-inflammatory and pro-angiogenic cytokines tumor necrosis factor (TNF)-α and IL-8 were released in THP-1 derived macrophages, which were stimulated by polymer capsules [61].

**Fig. 8:**
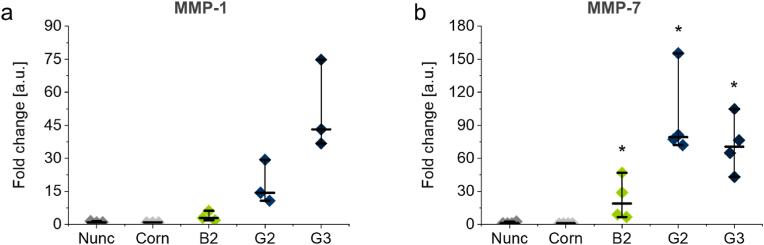
Release of matrix metalloproteinase (MMP)-1 (a) and MMP-7 (b) by moDCs cultured for 4 days on **B2** brush as well as **G2** and **G3** gel coatings and TCPS controls (Nunc, Corn). Protein levels were analyzed by ELISA in supernatants of moDCs generated from primary PBMCs of n = 3 (a) and n = 4 (b) healthy donors. Differences of MMP-1 and MMP-7 values were analyzed for statistical significance and compared to Nunc TCPS controls using the non-parametric Mann-Whitney-U test. Values of p < 0.05 were considered significant (*: *p* < 0.05).

For DCs in peripheral tissue, such as skin, it was reported that stimulation via the hepatocyte growth factor (HGF)/Met-signaling pathway could induce tolerogenic DCs [62,63]. HGF-activated DCs were mobilized and migrated accompanied by various phenotypic changes that include PD-L1 and matrix metalloproteinase (MMP) activation and detachment from surrounding tissue, as observed in wound healing [64]. Because *in vivo*-activated DCs can release various MMPs, we examined whether moDCs activated by PGE coatings also have the potential to release MMPs. Surprisingly, moDCs were found to secrete very high amounts of MMP-1 and MMP-7, up to 5 and 180 ng/ml per 10^6^ cells, respectively. MMP-9 was not detected (data not shown). Gel coatings **G3** and **G2** were found to be the most potent MMP inducers, while **B2** brush coatings still induced significantly elevated MMP-1 and MMP-7 levels compared to the Nunc TCPS control (Fig. 8). Similar to the expression of immune-inhibitory molecules, soluble PGEs **S1** and **S2** failed to induced high levels of MMP-1, -7, or -9 (Fig. S11). This indicates again that mechanical cues experienced by moDCs cultured on immobilized PGE coatings are essential for moDC activation accompanied by growth factor and MMP release [57].

In summary, PGE coatings constitute viable biomaterials for the intrinsic material-induced activation of moDCs. The elevated expression of cell surface markers CD86, CD40, and HLA-DR indicates that PGE-functionalized culture substrates activate moDCs substantially (Fig. 4, Fig. 5). Interestingly, activated moDCs showed simultaneous PD-L1 and -L2 expression and release of MMP-1 and -7 (Fig. 6, Fig. 8). This activation is especially pronounced for PGE gel coatings and seems to be rather independent on the GME:EGE comonomer ratio (1:1–1:7) of the gel-forming polymers but more dependent on their architecture on the surface, i.e., gel versus brush. General cell adhesion of immature moDCs, however, is hampered on thick gel coatings (15 nm) of the most hydrophilic copolymer with a GME:EGE comonomer ratio of 1:1, which can be improved by lowering the gel thickness to 2.5 nm. Further, PGE gels significantly promote the release of TGF-β1 and EGF (Fig. 7), which are important growth factors for tissue regeneration. Interestingly, PGE brushes exhibit a slightly less activating effect regarding the expression of cell surface markers and, especially, the release of growth factors. This attenuated moDC response is most likely due to the architecture and elasticity of the coatings with PGE brushes exhibiting lower thickness, more tightly packed polymer chain conformation as well as lower Young's modulus than PGE gels. In contrast, moDC activation is much more efficient on stiffer PGE gels, presumably since these gels present a higher amount of PGE material (Fig. 2a) with a rougher surface compared to PGE brushes and thus exhibit a higher surface area under culture conditions (Fig. 3). Further, differences in surface marker expression and growth factor release between **G2** and **G3** are negligible, with the exception of HLA-DR expression (Fig. 5c), even though these gel coatings have different compositions and, thus, differ in hydrophilicity as evident from water CAs (Fig. 2b). Our findings strongly suggest that the effect of PGE coatings is material-specific and, more specifically, that the PGE polymer structure induces the activation of moDCs. Importantly, this intrinsic activation is only observed when PGEs are tethered to a substrate and, thus, exert mechanical cues on the cultured immature moDCs. Hence, PGEs are very promising materials in the field of immunoengineering, particularly in two respects. As tissue culture substrates, PGE coatings are a potent candidate to facilitate the fabrication of immune-competent moDC-based *in vitro* tissue models. Secondly, PGE-functionalized biomaterial implants bear an intriguing potential for tissue reconstruction and remodeling. In addition, the therapeutic use of *in vitro*-activated moDCs might be realized in the future, however, it is important to note that the immune phenotype of moDCs activated in a 2D context can behave differently when reintroduced into the body's 3D tissues.

## 4 Conclusion

As a fully synthetic biomaterial, PGE-modified substrates demonstrated their potent capacity to activate human moDCs *in vitro*. In particular, PGE coatings enhance the expression of costimulatory and MHC-class-II molecules, PD-L1/-2 and MMP-1/-7 while simultaneously secreting growth factors TGF-β1 and EGF which are important players in tissue regeneration. The phenotypical and functional activation potency of moDCs by PGE coatings observed in this study bears great potential for the development of effective material-based tissue regeneration protocols, bioactive implant coatings, and clinical immunoengineering therapies. Further mechanistic investigations are currently underway to exploit the full potential of these materials and to identify the basic design guidelines for the fabrication of optimized PGE-based coatings.

## CRediT authorship contribution statement

**Daniel David Stöbener:** Methodology, Validation, Formal analysis, Investigation, Data curation, Writing – original draft, Writing – review & editing, Visualization. **Andrea Cosimi:** Methodology, Validation, Formal analysis, Investigation, Data curation, Visualization. **Marie Weinhart:** Conceptualization, Resources, Writing – review & editing, Supervision, Project administration, Funding acquisition. **Matthias Peiser:** Methodology, Validation, Formal analysis, Investigation, Data curation, Writing – original draft, Conceptualization, Resources, Writing – review & editing, Supervision, Project administration.

## Declaration of competing interest

The authors declare the following financial interests/personal relationships which may be considered as potential competing interests: Marie Weinhart, Matthias Peiser, and Daniel D. Stöbener have filed a patent METHOD FOR ACTIVATING DENDRITIC CELLS pending to Freie Universität Berlin.

## Data Availability

Data will be made available on request.
