# Supplementary material for: Efficient material-induced activation of monocyte-derived dendritic cells releasing surface molecules, matrix metalloproteinases, and growth factors needed for regenerative tissue remodeling
Source: Mater Today Bio. 2023 Nov 15;23:100869. doi: 10.1016/j.mtbio.2023.100869 (PMC10701458; doi:10.1016/j.mtbio.2023.100869)
Supplement: Multimedia component 1 [file mmc1.docx]

Supplementary material for the following research article submitted to *Materials Today Bio*

**Efficient Material-induced Activation of Monocyte-derived Dendritic Cells Releasing Surface Molecules, Matrix Metalloproteinases, and Growth Factors needed for Regenerative Tissue Remodeling**

*Daniel David Stöbener ^a,b^, Andrea Cosimi ^a,b^, Marie Weinhart ^a,b,^*, and Matthias Peiser ^c,^***

*^a^* Institute of Chemistry and Biochemistry - Organic Chemistry, Freie Universität Berlin, Takustr. 3, 14195 Berlin, Germany.

*^b^* Institute of Physical Chemistry and Electrochemistry, Leibniz Universität Hannover, Callinstr. 3A, 30167 Hannover, Germany.

*^c^* Institute of Chemistry and Biochemistry - Biochemistry, Freie Universität Berlin, Thielallee 63, 14195, Berlin, Germany.

1. **Materials and methods**

*Polymer Synthesis:* Block and statistical PGE terpolymers comprising photo-reactive BP moieties were synthesized using the monomer-activated anionic ring-opening polymerization (MA-AROP). PGE block copolymers **B1** and **B2** with EBP-based anchor blocks were obtained via the sequential MA-AROP as reported previously (**Fig. S1a**, **Table S1**) [1, 2]. Statistical PGE terpolymers **G1**, **G2**, and **G3** with BP units along the polymer backbone were synthesized via a two-step post-modification protocol of allyl-functional terpolymers based on our previous reports (**Fig. S1b**, **Table S1**) [2-4]. The detailed synthesis of statistical PGE terpolymers is described further below. Random PGE copolymers **S1** and **S2** without photo-reactive BP units serving as soluble controls for moDC activation were synthesized according to established procedures (**Fig. S1c**, **Table S1**) [3, 5, 6]. For polymer characterization, ^1^H NMR spectra were recorded using CDCl_3_ as a solvent on a Joel ECX at 500 MHz and processed with the software MestReNova (version 7.1.2). Gel permeation chromatography (GPC) was conducted on an Agilent 1100 Series instrument in THF as the eluent at concentrations of 3.5 mg mL^-1^ and a flow rate of 1 mL min^-1^ at 25 °C. Three PLgel mixed-C columns (Agilent, Waldbronn, Germany) with dimensions of 7.5 × 300 mm and a particle size of 5 µm were used in line with a refractive index detector. Calibration was performed with polystyrene (PS) standards from PSS (Mainz, Germany), and calculation was performed with PSS Win-GPC software.


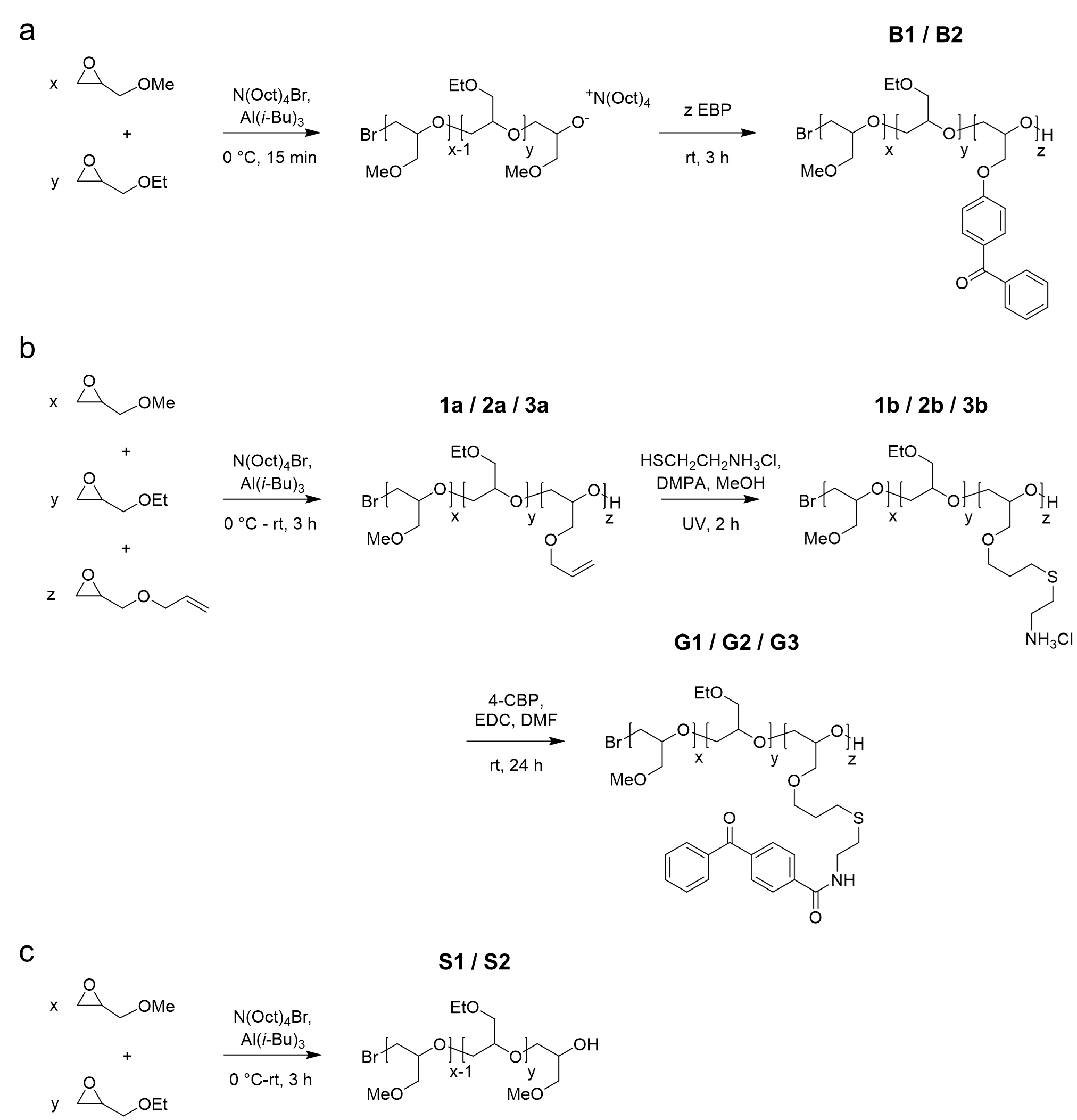


**Fig. S1.** (a) Schematic synthesis of poly(GME-*ran.*-EGE)-*block*-poly(EBP) block copolymers **B1** and **B2** via the sequential MA-AROP. (b) Schematic synthesis of poly(GME‑*stat.*‑EGE‑*stat.*-AGE) terpolymers **1a**, **2a**, and **3a** via the MA-AROP and their 2-step post-modification to poly(GME-*stat.*-EGE-*stat.*-AC-BP) to **G1**, **G2**, and **G3** via photo-chemical thiol-ene coupling of cysteamine hydrochloride and subsequent amidation with 4‑benzoylbenzoic acid (4‑CBP). (c) Schematic synthesis of poly(GME-*ran.*-EGE) copolymers **S1** and **S2** via the MA-AROP of GME and EGE.

**Table S1.** Theoretical and experimental molecular weight data and comonomer compositions of PGE block copolymers **B1** and **B2**, statistical PGE terpolymers **G1**, **G2**, and **G3**, and random PGE copolymers **S1** and **S2** determined by gel permeation chromatography (GPC) and proton nuclear magnetic resonance (^1^H NMR) spectroscopy.

| PGE | *M*_n, theor._ [kDa] | *M*_n_***_, GPC_ [kDa] | PDI*_GPC_ | GME:EGE  (theor.) | GME:EGE  (NMR) | BP units  (theor.) | BP units  (NMR) |
| --- | --- | --- | --- | --- | --- | --- | --- |
| B1 | 30 | 28.4 | 1.21 | 1:1 | 1.1 : 1.0 | 5.0 | 4.2 |
| B2 | 30 | 27.1 | 1.20 | 1:3 | 1.0 : 2.8 | 5.0 | 4.8 |
| G1 | 40 | 47.5 | 1.19 | 1:1 | 1.0 : 1.0 | 6.3 | 5.0 |
| G2 | 40 | 45.1 | 1.28 | 1:3 | 1.0 : 2.8 | 6.1 | 4.9 |
| G3 | 40 | 46.9 | 1.25 | 1:7 | 1.0 : 6.0 | 6.0 | 4.4 |
| S1 | 40 | 42.5 | 1.27 | 1:1 | 1.0 : 1.0 | N/A | N/A |
| S2 | 40 | 40.4 | 1.20 | 3:1 | 3.2 : 1.0 | N/A | N/A |

*number average molecular weight (*M*_n_) and polydispersity index (PDI) as determined by GPC in tetrahydrofuran applying polystyrene standards; N/A: not applicable.

*Synthesis of statistical PGE terpolymers:* Statistical poly(GME-*stat.*-EGE-*stat.*-AC-BP) terpolymers **G1**, **G2**, and **G3** were synthesized via the MA-AROP and a subsequent 2-step post modification via thiol-ene chemistry and amidation according to a previously reported procedure with slight modification [2]. First, allyl-functional poly(GME-*stat.*-EGE-*stat.*-AGE) terpolymers were synthesized according to the following general procedure. The initiator tetraoctylammonium bromide (N(Oct)_4_Br, 1 eq) was melted and dried in a flame-dried Schlenk flask (100 mL) under high vacuum at 103 °C and dissolved in dry toluene (monomer concentration = 1.5 M) at room temperature. The solution was cooled to 0 °C with an ice bath, and the dry monomers GME, EGE, and AGE were added under an inert gas atmosphere via a syringe in the respective targeted ratio. The polymerization was initiated via the rapid addition of the activator triisobutyl aluminum (Al(*i*‑Bu)_3_, 5 eq) in anhydrous hexane (1 M) and stirred for 3 h while being allowed to warm up to room temperature. The reaction was quenched by adding Milli-Q water (~ 0.5 mL), stirred for 1 h, dried over Na_2_SO_4_ for 1 h under stirring, and subsequently filtered. After removing toluene under reduced pressure, the crude polymers were dissolved in Et_2_O (40 ml), and residual initiator salts were precipitated by centrifugation at 0 °C. After decanting, Et_2_O was evaporated from the supernatants, and the remaining terpolymers were dissolved and dialyzed against MeOH with regenerated cellulose dialysis tubings (Spectra Pore, MWCO 1 kDA) for 3 d and obtained as pale yellow viscous oils.

Poly(GME-*stat.*-EGE-*stat.*-AGE) (**1a**):

N(Oct)_4_Br = 172 mg (0.32 mmol, 1 eq), GME = 4.50 mL (50.15 mmol, 210.3 eq), EGE = 5.45 mL (50.15 mmol, 210.3 eq), Al(*i*-Bu)_3_ (1 M in hexanes) = 1.2 mL (1.2 mmol, 5 eq), AGE = 0.18 mL (1.51 mmol, 6.3 eq), toluene = 65 mL, yield = 96%; ^1^H NMR (500 MHz; CDCl_3_): δ(ppm) = 5.87 (m, -OCH_2_CHCH_2_); 5.27-5.13 (m, ‑OCH_2_CHCH_2_); 3.98-3.97 (m, -OCH_2_CHCH_2_); 3.61-3.47 (m, polymer backbone, -OCH_2_CH_3_); 3.33 (s, -OCH_3_); 1.17 (t, -OCH_2_CH_3_); GPC: *M*_n_ = 47.5 kg mol^-1^, PDI = 1.19.

Poly(GME-*stat.*-EGE-*stat.*-AGE) (**2a**):

N(Oct)_4_Br = 138 mg (0.25 mmol, 1 eq), GME = 2.30 mL (25.63 mmol, 101.3 eq), EGE = 8.40 mL (76.90 mmol, 304.0 eq), Al(*i*-Bu)_3_ (1 M in hexanes) = 1.27 mL (1.27 mmol, 5 eq), AGE = 0.18 mL (1.54 mmol, 6.1 eq), toluene = 65 mL, yield = 94%; ^1^H NMR (500 MHz; CDCl_3_): δ(ppm) = 5.87 (m, -OCH_2_CHCH_2_); 5.27-5.13 (m, -OCH_2_CHCH_2_); 3.98-3.97 (m, -OCH_2_CHCH_2_); 3.62-3.47 (m, polymer backbone + -OCH_2_CH_3_); 3.33 (s, -OCH_3_); 1.17 (t, -OCH_2_CH_3_); GPC: *M*_n_ = 45.1 kg mol^-1^, PDI = 1.28.

Poly(GME-*stat.*-EGE-*stat.*-AGE) (**3a**):

N(Oct)_4_Br = 135 mg (0.25 mmol, 1 eq), GME = 1.1 mL (12.3 mmol, 49.8 eq), EGE = 9.3 mL (85.8 mmol, 348.9 eq), Al(*i*-Bu)_3_ (1 M in hexanes) = 1.23 mL (1.23 mmol, 5 eq), AGE = 0.18 mL (1.47 mmol, 6.0 eq), toluene = 65 mL, yield = 96%; ^1^H NMR (500 MHz; CDCl_3_): δ(ppm) = 5.87 (m, -OCH_2_CHCH_2_); 5.27-5.13 (m, -OCH_2_CHCH_2_); 3.98-3.97 (m, -OCH_2_CHCH_2_); 3.61-3.47 (m, polymer backbone + -OCH_2_CH_3_); 3.33 (s, -OCH_3_); 1.17 (t, -OCH_2_CH_3_); GPC: *M*_n_ = 46.9 kg mol^-1^, PDI = 1.25.

Poly(GME-*stat.*-EGE-*stat.*-AGE) terpolymers **1a**, **2a**, **3a** equipped with statistically distributed AGE-based allyl units were functionalized with photo-reactive BP units via a two-step post-polymerization protocol according to our previous report [2]. In the first step, the allyl groups were functionalized with cysteamine groups via thio-ene chemistry. In brief, PGE terpolymers **1a**, **2a**, or **3a** (1 eq), 2-aminoethanethiol hydrochloride (5 eq), and the photo-initiator 2,2‑dimethoxy-2-phenylacetophenone (DMPA, 0.2 eq) were dissolved in MeOH (10 mL) in a 50 mL reaction vial and purged with Ar for 15 min while protected against light. The reaction mixtures were then irradiated with broad-spectrum UV light using a 150 W Hg arc lamp from Quantum Design GmbH (Darmstadt, Germany) at 90 W (60% power) for 2 h. The crude products were dialyzed (Spectra Pore, MWCO 1 kDA) against MeOH for 3 d. After removing the solvent under reduced pressure, poly(GME‑*stat.*‑EGE‑*stat.*‑AC) terpolymers were obtained as pale yellow liquids.

Poly(GME-*stat.*-EGE-*stat.*-AC) (**1b**):

**1a** = 2 g (6.3 AGE/chain, 1 eq), 2-aminoethanethiol hydrochloride = 254 mg (2.24 mmol, 5 eq), DMPA = 23 mg (0.09 mmol, 0.2 eq), MeOH = 10 mL, conversion (AGE) = 84%, yield = 90%; ^1^H NMR (500 MHz; CDCl_3_): δ(ppm) = 3.62-3.42 (m, polymer backbone + -OCH_2_CH_3_ + -OCH_2_CH_2_CH_2_S-); 3.34 (s, -OCH_3_); 3.12 (m, -SCH_2_CH_2_NH_2_); 2.94 (m, -SCH_2_CH_2_NH_2_); 2.68 (m, -OCH_2_CH_2_CH_2_S-); 1.85 (m, -OCH_2_CH_2_CH_2_S-); 1.17 (t, -OCH_2_CH_3_).

Poly(GME-*stat.*-EGE-*stat.*-AC) (**2b**):

**2a** = 2 g (6.1 AGE/chain, 1 eq), 2-aminoethanethiol hydrochloride = 269 mg (2.37 mmol, 5 eq), DMPA = 24 mg (0.09 mmol, 0.2 eq), MeOH = 10 mL, conversion (AGE) = 84%, yield = 93%; ^1^H NMR (500 MHz; CDCl_3_): δ(ppm) = 3.61-3.42 (m, polymer backbone + -OCH_2_CH_3_ + -OCH_2_CH_2_CH_2_S-); 3.33 (s, -OCH_3_); 3.12 (m, -SCH_2_CH_2_NH_2_); 2.94 (m, -SCH_2_CH_2_NH_2_); 2.68 (m, -OCH_2_CH_2_CH_2_S-); 1.85 (m, -OCH_2_CH_2_CH_2_S-); 1.17 (t, -OCH_2_CH_3_).

Poly(GME-*stat.*-EGE-*stat.*-AC) (**3b**):

**3a** = 2 g (6.0 AGE/chain, 1 eq), 2-aminoethanethiol hydrochloride = 254 mg (2.24 mmol, 5 eq), DMPA = 23 mg (0.09 mmol, 0.2 eq), MeOH = 10 mL, conversion (AGE) = 80%, yield = 92%; ^1^H NMR (500 MHz; CDCl_3_): δ(ppm) = 3.62-3.42 (m, polymer backbone + -OCH_2_CH_3_ + -OCH_2_CH_2_CH_2_S-); 3.34 (s, -OCH_3_); 3.12 (m, -SCH_2_CH_2_NH_2_); 2.94 (m, -SCH_2_CH_2_NH_2_); 2.68 (m, -OCH_2_CH_2_CH_2_S-); 1.85 (m, -OCH_2_CH_2_CH_2_S-); 1.17 (t, -OCH_2_CH_3_).

In the second post-functionalization step, the amine group bearing PGE terpolymers **1b**, **2b**, and **3b** were functionalized with benzophenone (BP) groups via amide coupling. In brief, to polymer **1b**, **2b**, or **3b** and 4-benzoylbenzoic acid (4-CBP, 5 eq) solutions in dry DMF (10 mL) in a 100 mL round bottom flask under inert gas atmosphere were added solutions of *N*‑(3‑dimethylaminopropyl)-*N*′-ethylcarbodiimide hydrochloride (EDC-HCl, 5 eq) in dry DMF (5 mL). The mixtures were stirred at room temperature for 24 h. The crude product solutions were diluted with MeOH (20 mL) and dialyzed (Spectr Pore, MWCO 1kDA) against MeOH for 3 d. After removing the solvent under reduced pressure, **G1**-**G3** were obtained as highly viscous pale-yellow liquids.

Poly(GME-*stat.*-EGE-*stat.*-AC-BP) (**G1**):

**1b** = 1.5 g (5.3 NH_2_/chain, 1 eq), 4-CBP = 318 mg (1.41 mmol, 5 eq), EDC‑HCl = 270 mg (1.41 mmol, 5 eq), DMF = 15 mL, conversion (NH_2_) = 94%, yield = 96%; ^1^H NMR (500 MHz; CDCl_3_): δ(ppm) = 7.91 (m, 2H, BP); 7.79-7.75 (m, 4H, BP); 7.58 (m, 1H, BP); 7.46 (m, 2H, BP); 3.61-3.42 (m, polymer backbone + -OCH_2_CH_3_ + -OCH_2_CH_2_CH_2_S- + -SCH_2_CH_2_NHCOBP); 3.33 (s, -OCH_3_); 2.77 (m, -SCH_2_CH_2_NHCOBP); 2.62 (m, -OCH_2_CH_2_CH_2_S-); 1.82 (m, -OCH_2_CH_2_CH_2_S-); 1.16 (t, -OCH_2_CH_3_).

Poly(GME-stat.-EGE-stat.-AC-BP) (**G2**):

**2b** = 1.5 g (5.1 NH_2_/chain, 1 eq), 4-CBP = 312 mg (1.38 mmol, 5 eq), EDC‑HCl = 265 mg (1.38 mmol, 5 eq), DMF = 15 mL, conversion (NH_2_) = 96%, yield = 97%; ^1^H NMR (500 MHz; CDCl_3_): δ(ppm) = 7.91 (m, 2H, BP); 7.79-7.75 (m, 4H, BP); 7.58 (m, 1H, BP); 7.46 (m, 2H, BP); 3.61-3.42 (m, polymer backbone + -OCH_2_CH_3_ + -OCH_2_CH_2_CH_2_S- + -SCH_2_CH_2_NHCOBP); 3.33 (s, -OCH_3_); 2.77 (m, -SCH_2_CH_2_NHCOBP); 2.62 (m, -OCH_2_CH_2_CH_2_S-); 1.82 (m, -OCH_2_CH_2_CH_2_S-); 1.16 (t, -OCH_2_CH_3_).

Poly(GME-*stat.*-EGE-*stat.*-AC-BP) (**G3**):

**3b** = 1.5 g (4.8 NH_2_/chain, 1 eq), 4-CBP = 318 mg (1.41 mmol, 5 eq), EDC‑HCl = 270 mg (1.41 mmol, 5 eq), DMF = 15 mL, conversion (NH_2_) = 92%, yield = 95%; ^1^H NMR (500 MHz; CDCl_3_): δ(ppm) = 7.91 (m, 2H, BP); 7.79-7.75 (m, 4H, BP); 7.58 (m, 1H, BP); 7.46 (m, 2H, BP); 3.61-3.42 (m, polymer backbone + -OCH_2_CH_3_ + -OCH_2_CH_2_CH_2_S- + -SCH_2_CH_2_NHCOBP); 3.33 (s, -OCH_3_); 2.77 (m, -SCH_2_CH_2_NHCOBP); 2.62 (m, -OCH_2_CH_2_CH_2_S-); 1.82 (m, -OCH_2_CH_2_CH_2_S-); 1.16 (t, -OCH_2_CH_3_).

*Methods for Surface Preparation and Characterization:* Spin-coating was performed using a WS-650-23 spin-coater from Laurell Technologies (North Wales, PA, USA). Si wafer model substrates (11 x 11 mm) were coated at 3000 rpm for 60 s using 50 µL of a 1% (w/w) solution of PS (derived from Falcon^®^ PS suspension culture dishes) in toluene. PGE gel coatings were spin-coated at 3000 rpm for 60 s using 50 µL for PS-coated Si wafers and 100 µL for PS dishes of a 1% (w/w) solution of PGEs **G1**-**G3** in EtOH. For photo-immobilization, the PGE-functionalized surfaces were irradiated with UV light using a UV-KUB 2 (λ = 365 nm, irradiance = 25 mW cm^‑2^) from Kloé (Montpellier, France) for 160 s corresponding to a radiant exposure of 4.0 J cm^‑2^. Static water contact angles (CAs) were measured with an OCA contact angle system from DataPhysics Instruments GmbH (Filderstadt, Germany) and fitted with the software package SCA202 (version 3.12.11) using the sessile drop method. CAs were determined before and after surface functionalization under ambient conditions at 20 °C. A drop of Milli-Q water (2 μL) was placed onto the respective surface, and CAs were determined with the Young-Laplace model. For each substrate, CAs were measured on at least five different spots to test for the homogeneity of the sample and at least six independent substrates (n = 6) to test for reproducibility. The dry layer thickness of the polymer coatings was determined by spectroscopic ellipsometry (SE) at an incident angle of 70° with a SENpro spectroscopic ellipsometer from Sentech Instruments GmbH (Berlin, Germany). The thickness of the SiO_2_ layer before spin coating and the additional thickness of the spin-coated PS layers were determined separately using a Cauchy layer for modeling, and respective average values of at least five different spots on the surfaces were taken as fixed values for the subsequent modeling of the PGE layers. The PGE thickness was measured at wavelengths from 370 nm to 1050 nm and was fitted using a model consisting of the previously measured layers with fixed parameters, a PGE layer with a fixed refractive index of n = 1.45, and air as the surrounding medium. AFM measurements were conducted using a Nanoscope Multimode 8 device equipped with a fluid cell and a thermal application controller from Bruker (Billerica, MA, USA). The morphology and mechanical properties of the PGE coatings were measured in Quantitative Nanomechanical Mapping (QNM) mode. Therefore, PGE-coated Si wafers model substrates were mounted on the AFM head, degassed Milli‑Q water was inserted into the liquid cell, and the TA controller was set to 37 °C and equilibrated for at least 10 min before each measurement. To obtain high-resolution images with reduced sample damage, SNL-10A cantilevers from Bruker (Billerica, MA, USA) with a nominal spring constant of 0.3 N m^-1^ and a tip radius of 2-12 nm were used, and images were recorded with a loading peak force of 1 nN, 512 points per line, and scan rates of 1.0 Hz. The sensitivity and spring constant of the cantilevers were determined separately before each measurement using the thermal noise method [7, 8]. Obtained images were analyzed with the Nanoscope analysis software (version 1.4) and processed using 1^st^ order flattening. Roughness and depth analysis tools were used to obtain the respective surface parameters. Additional AFM nanoindentation measurements with colloidal probes were performed with a JPK NanoWizard 4 from Bruker Nano GmbH (Berlin, Germany). Colloidal probes were prepared by gluing SiO_2_ beads (*d* = 4.77 ± 0.20 µm) (Lot: SiO_2_-F-KM358) from Microparticles GmbH (Berlin, Germany) to tipless silicon Arrow-TL1-50 cantilevers (resonance frequency: 6 kHz; nominal spring constant (k): 0.03 N m-1) from Nanoworld AG (Neuchâtel, Switzerland) with a fast-curing (~ 8 min), two-component epoxy glue from Bindulin H.L. Schoenleber GmbH (Fuerth, Germany). The measurements were performed in contact mode (force spectroscopy) with z‑closed loop. The relative setpoint was set to 1 or 0.5 nN with a Z-length of 5-15 µm, an extension speed of 5-20 µm/s, and a sample rate of 3000 Hz (2225 pixels). Raw data were processed with JPKSPM Data Processing software (v. 6.1.198.).

1. **Surface characterization via AFM**

A comparison between the morphology and mechanical properties of **B2** and **G2** coatings measured by QNM is illustrated in **Fig. S2** and **Fig. S3**, respectively. As indicated by the deformation maps and histograms in **Fig. S3a-c**, **G2** gels exhibit a higher deformation than **B2** brushes at comparable copolymer composition (GME:EGE ~ 1:3). Considering that the same indentation force was applied on both surfaces, this is also reflected in a lower elastic modulus of **G2**, as illustrated in **Fig. S3d-f**. Both, deformation and elastic moduli, affirm the difference between the brush- and gel-type architectures of the two coatings, respectively. Furthermore, the higher adhesive forces between **G2** gels and the AFM tip, which are depicted and compared to **B2** brushes in **Fig. S3g-i**, indicate a more pronounced interaction of the PGE **G2** gel with the Si_3_N_4_ AFM tip. In general, all investigated brush and gel coatings, respectively, exhibit comparable mechanical properties which are largely independent of their comonomer composition within the investigated range (GME:EGE = 1:1-1:7). Comparisons between the morphology and the mechanical properties of PGE brush and gel coatings with different comonomer compositions, respectively, are given in **Fig. S4-S7** and are in line with the results from the presented **B2** brush versus **G2** gel comparison (**Fig. S2-S3**). However, since the underlying hard PS substrate strongly influences the absolute values of the elastic moduli of such thin coatings, complementary nanoindentation measurements with a colloidal probe have been performed. Due to the larger contact area of colloidal SiO_2_ probes (*d* ~ 4.8 µm) compared to a pyramidal Si_3_N_4_ tip (*r* ~10 nm), more realistic, absolute Young’s modulus values, which largely exclude the substrate bias, were obtained (**Fig. S8**). Contrary to QNM results, colloidal probe nanoindentation measurements indicate that the crosslinked PGE gels have at least 2-3 times higher Young’s moduli than PGE brushes due to their stiffer network structure. These significant and meaningful differences are only detectable in the absence of the substrate bias and at a sufficiently high indentation peak force setpoint of 1 nN (**Fig. S8a, b**). At a lower peak force setpoint of 0.5 nN, Young’s moduli are consistently lower and only slightly higher for PGE gels than brushes (**Fig. S8c, d**). This is due to the insufficient deformation of the gel coatings, which leads to the measurement of the topmost gel layer comprising less crosslinked and, thus, softer dangling polymer chains.


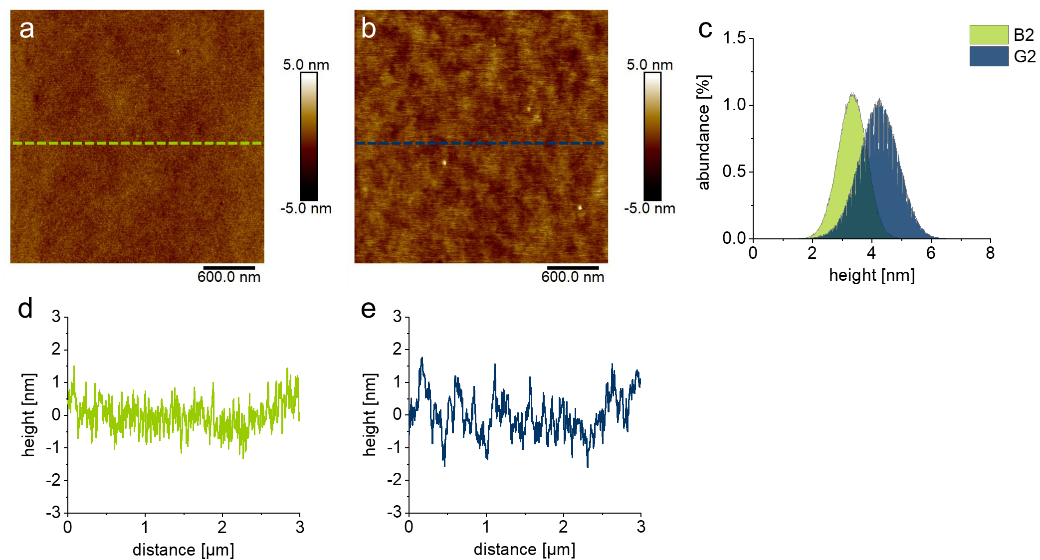


**Fig. S2.** Representative morphological images of PGE brush **B2** (a) and gel **G2** (b) coatings on PS-coated silicon wafer model substrates, corresponding height depth histograms (c), and respective height cross-section profiles (d, e) measured in water at 37 °C via AFM.


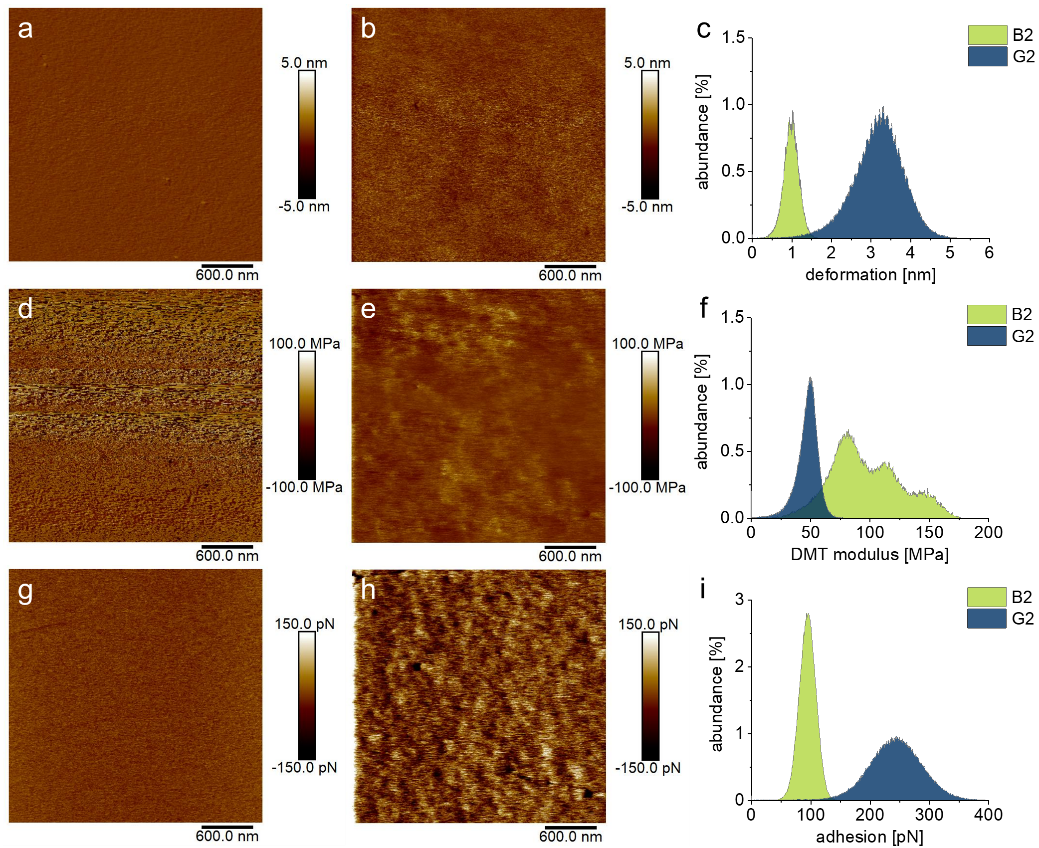


**Fig. S3.** Representative deformation (a-c), elastic modulus (d-f), and adhesion (g-i) images and respective comparative depth histograms (c, f, i) of PGE brush **B2** (a, d, g) and gel **G2** (b, e, h) coatings measured in water at 37 °C via AFM.


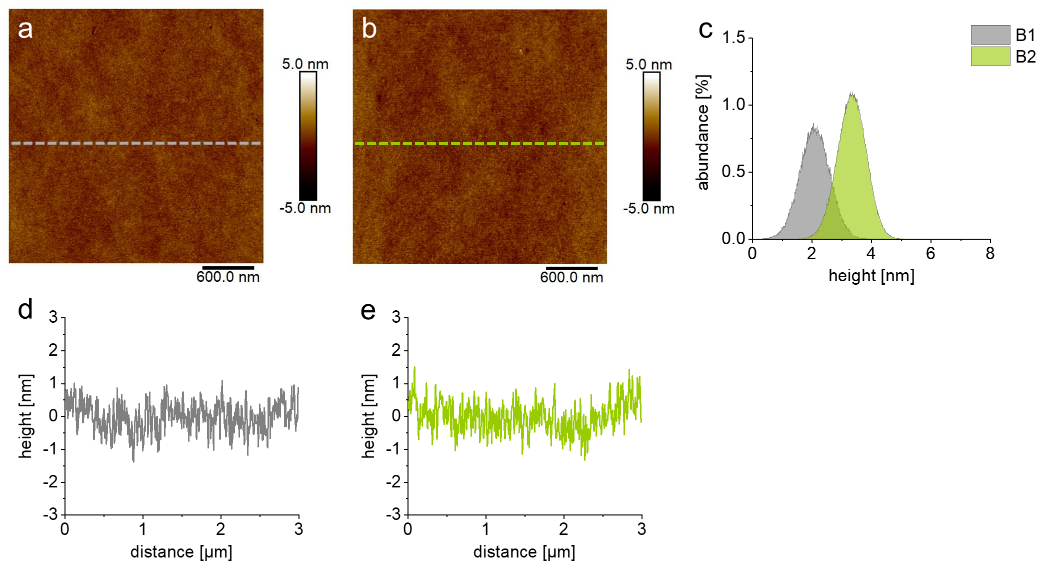


**Fig. S4.** Representative morphological images of PGE brush **B1** (a) and **B2** (b) coatings on PS-coated silicon wafer model substrates, corresponding height depth histograms (c), and respective height cross-section profiles (d, e) measured in water at 37 °C via AFM.


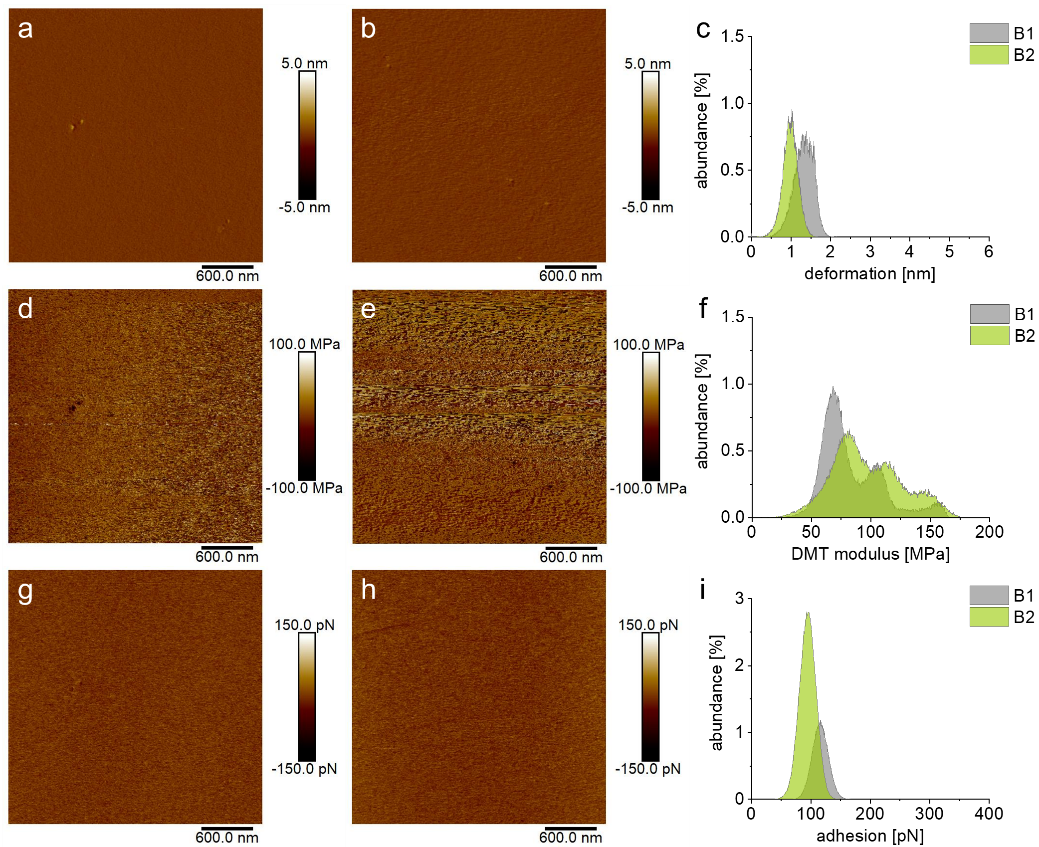


**Fig. S5**. Representative deformation (a-c), elastic modulus (d-f), and adhesion (g-i) images and respective comparative depth histograms of PGE brush **B1** (a, d, g) and **B2** (b, e, h) coatings measured in water at 37 °C via AFM.


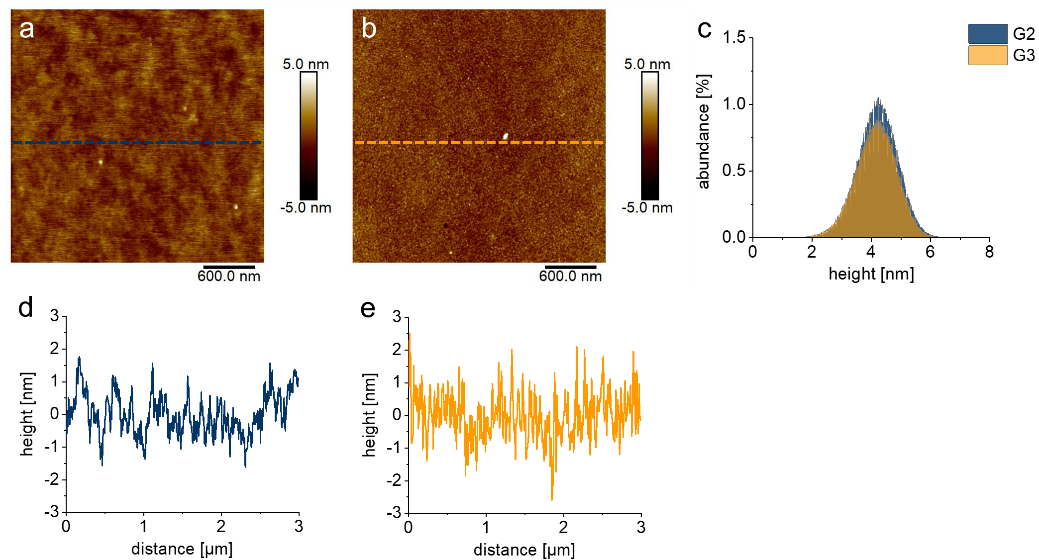


**Fig. S6**. Representative morphological images of PGE gel **G2** (a) and **G3** (b) coatings on PS-coated silicon wafer model substrates, corresponding height depth histograms (c), and respective height cross-section profiles (d, e) measured in water at 37 °C via AFM.


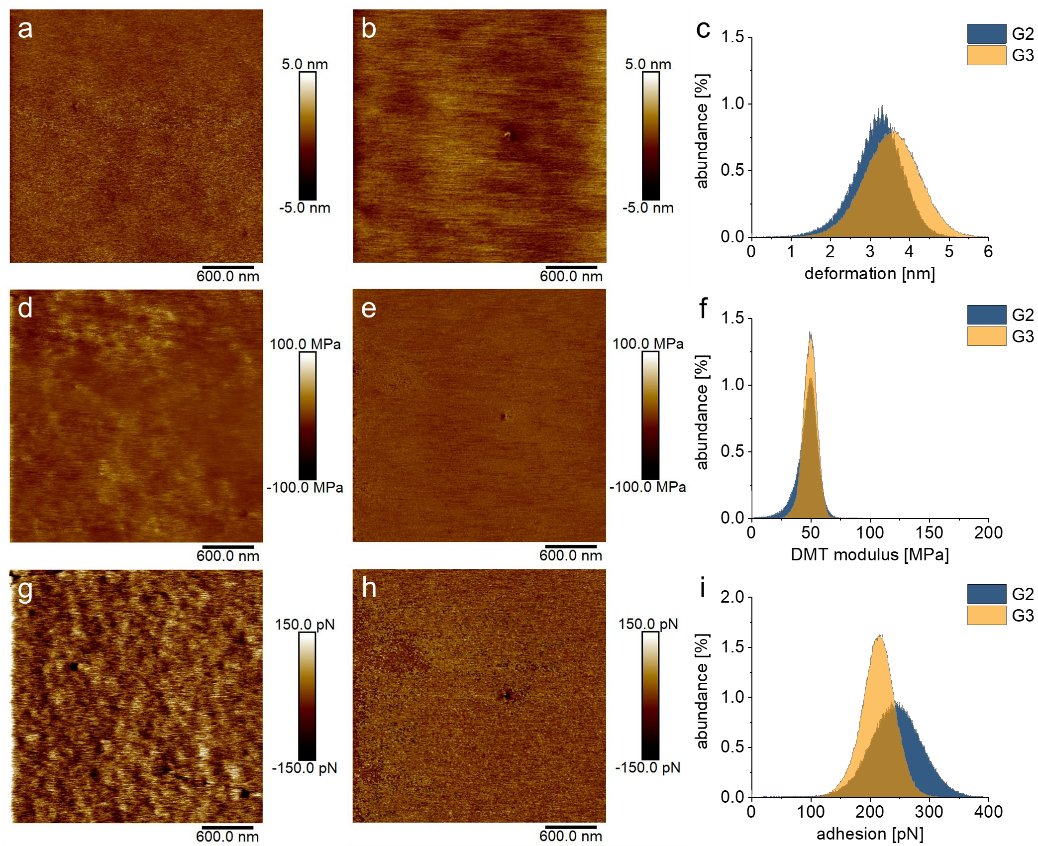


**Fig. S7**. Representative deformation (a-c), elastic modulus (d-f), and adhesion (g-i) images and respective comparative depth histograms of PGE gel **G2** (a, d, g) and **G3** (b, e, h) coatings measured in water at 37 °C via AFM.


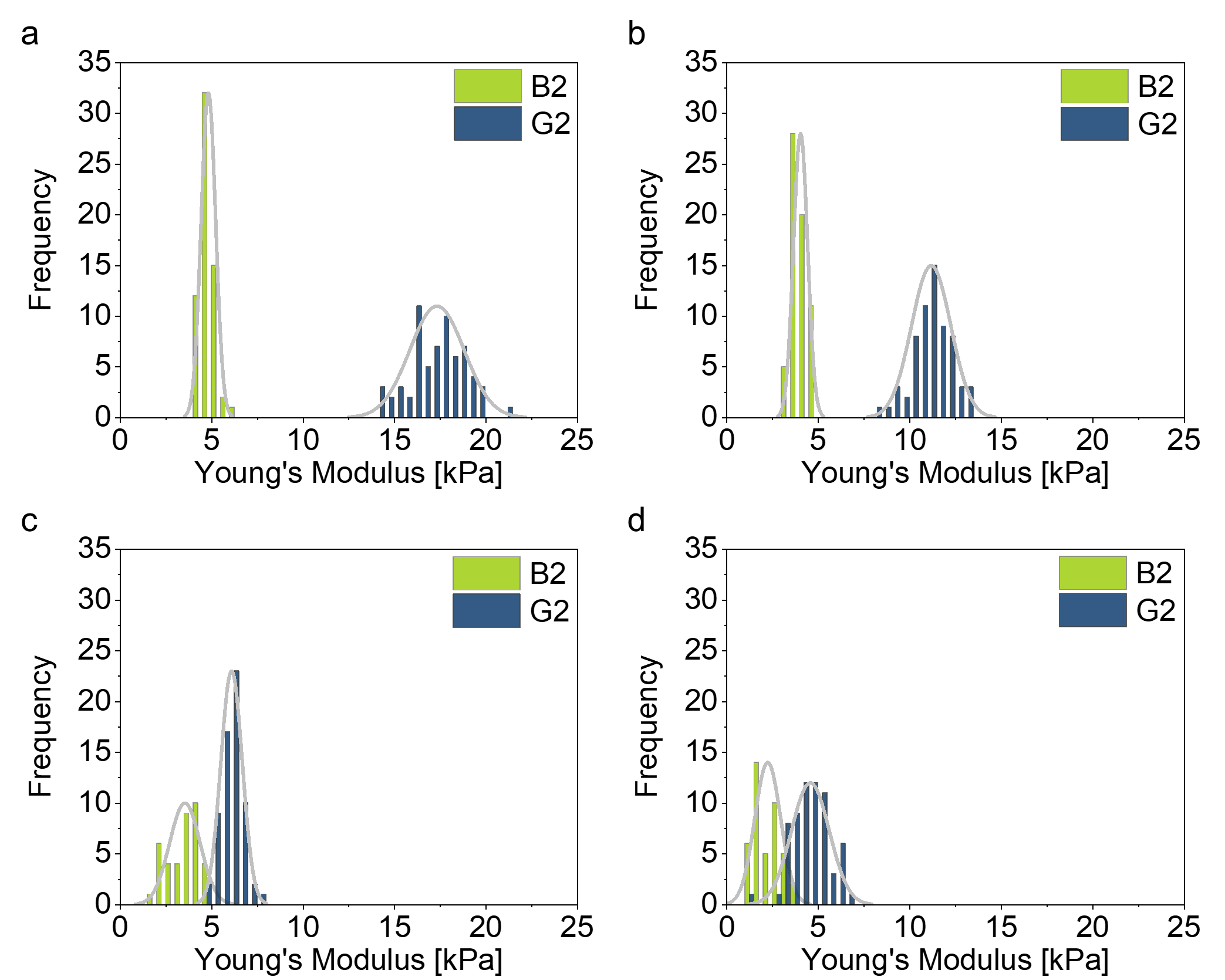


**Fig. S8**. Representative comparative Young’s moduli of **B2** brush and **G2** gel coatings on PS-coated Si wafer model substrates obtained by AFM nanoindentation measurements using a colloidal SiO_2_ probe (*d* ~ 4.8 µm) in water at 37 °C. (a) Setpoint = 1 nN, z-length = 10 µm, speed = 10 µm s^-1^; (b) Setpoint = 1 nN, z-length = 10 µm, speed = 20 µm s^-1^; (c) Setpoint = 0.5 nN, z-length = 5 µm, speed = 5 µm s^-1^; (d) Setpoint = 0.5 nN, z‑length = 5 µm, speed = 10 µm s^-1^;

1. **Endotoxin test**

The potential presence of liposaccharides (LPS) on PGE coatings was tested using a Pierce™ LAL chromogenic endotoxin quantitation kit from Thermo Fisher Scientific (Darmstadt, Germany) according to the manufacturer’s instruction. Calibration curves were recorded with an Infinite 200 PRO microplate reader from Tecan Trading AG (Switzerland) at 405 nm (**Fig. S9a**). Endotoxin levels were measured on PGE gel coatings **G2** and **G3** at varying thicknesses (5-30 nm) in order to assess whether the polymeric raw materials contain DC-activating LPS. Bare PS culture dishes (B), as well as culture dishes that were run through the coating procedure without the presence of PGEs **G2** and **G3** (C), were used as controls. As shown in **Fig. S9b**, endotoxin levels were in the range of the lower detection limit of bare (B) and pseudo-coated (C) control substrates. The activation of moDCs by abundant endotoxins, i.e., LPS, on the coated surfaces is therefore excluded.


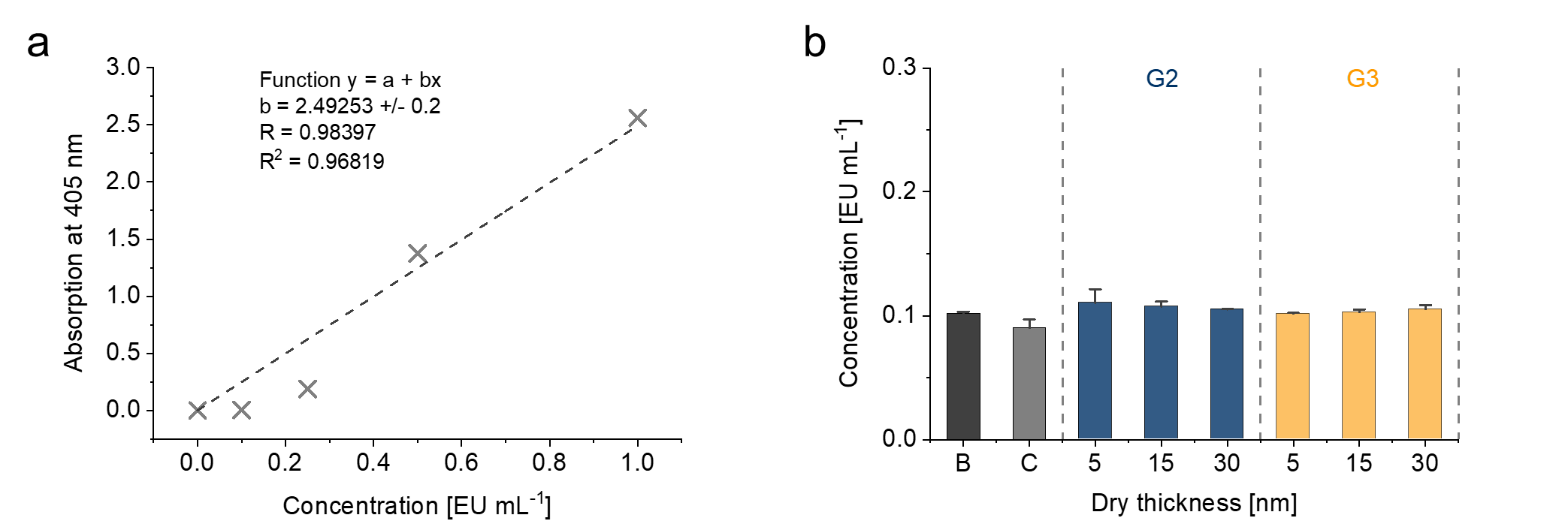


**Fig. S9.** (a) Calibration curve for the colorimetric endotoxin test plotted as absorption at 405 nm versus LPS concentration in EU mL^-1^. (b) Endotoxin levels determined for blank (B) and pseudo-coated (C) control substrates and **G2** as well as **G3** gel coatings at different dry layer thicknesses (5, 15, and 30 nm).

1. **MoDC activation by soluble PGE copolymers**

The activation potential of soluble PGE copolymers **S1** and **S2** (**Fig. S1c**, **Table S1**) was compared to **B2** brush as well as **G2** and **G3** gel coatings to deduce whether surface immobilization is a prerequisite for moDC instruction. Therefore, solutions of **S1** and **S2** were prepared in supplemented RPMI 1640 culture medium (see METHODS) at a concentration of 10 µg mL^-1^. At this concentration, the amount of dissolved PGE is comparable to that of immobilized PGE material within 15 nm thick gel coatings, thus serving as an appropriate control. PGE stock solutions (250 µg mL^-1^) were prepared by dissolving 10 mg copolymer in 40 mL medium. 2 mL of the respective stock solutions were then diluted with 48 mL medium and subsequently sterile filtered at 20 °C under clean bench conditions. The PGE-containing media were then used to culture moDCs in conventional TCPS dishes. It is important to note that **S1** with a GME:EGE comonomer ratio of 1:1 (**Table S1**) predominantly forms multimolecular particles in the higher nanometer range (~ 100 nm) under standard culture conditions at 37 °C. This is due to the copolymer’s thermoresponsive lower critical solution temperature (LCST)-type phase transition behavior which induces intermolecular polymer chain association into mesoglobular aggregates above ~ 30 °C [2]. In contrast, **S2** with a more hydrophilic GME:EGE comonomer composition of 3:1 (**Table S1**) exhibits its phase transition temperature > 40 °C (data not shown) and is therefore fully dissolved under standard culture conditions at 37 °C presenting monomolecularly dissolved, individual polymer chains.

As illustrated in **Fig. S10**, CD54 positive moDCs cultured in the presence of soluble PGE copolymers **S1** and **S2** do not express elevated levels of immune-control associated molecules PD-L1 and -L2 as compared to TCPS controls and, accordingly, lower levels than moDCs cultured on **B2**, **G2**, and **G3** coatings. Similarly, the release of MMP-1 and -7 was not significantly elevated in moDCs cultured in the presence of **S1** and **S2** as compared to moDCs cultured on **B2**, **G2**, and **G3** coatings (**Fig. S11**). These results strongly suggest that surface immobilization is a necessity to obtain PGE-based biomaterials with moDC-instructive properties.


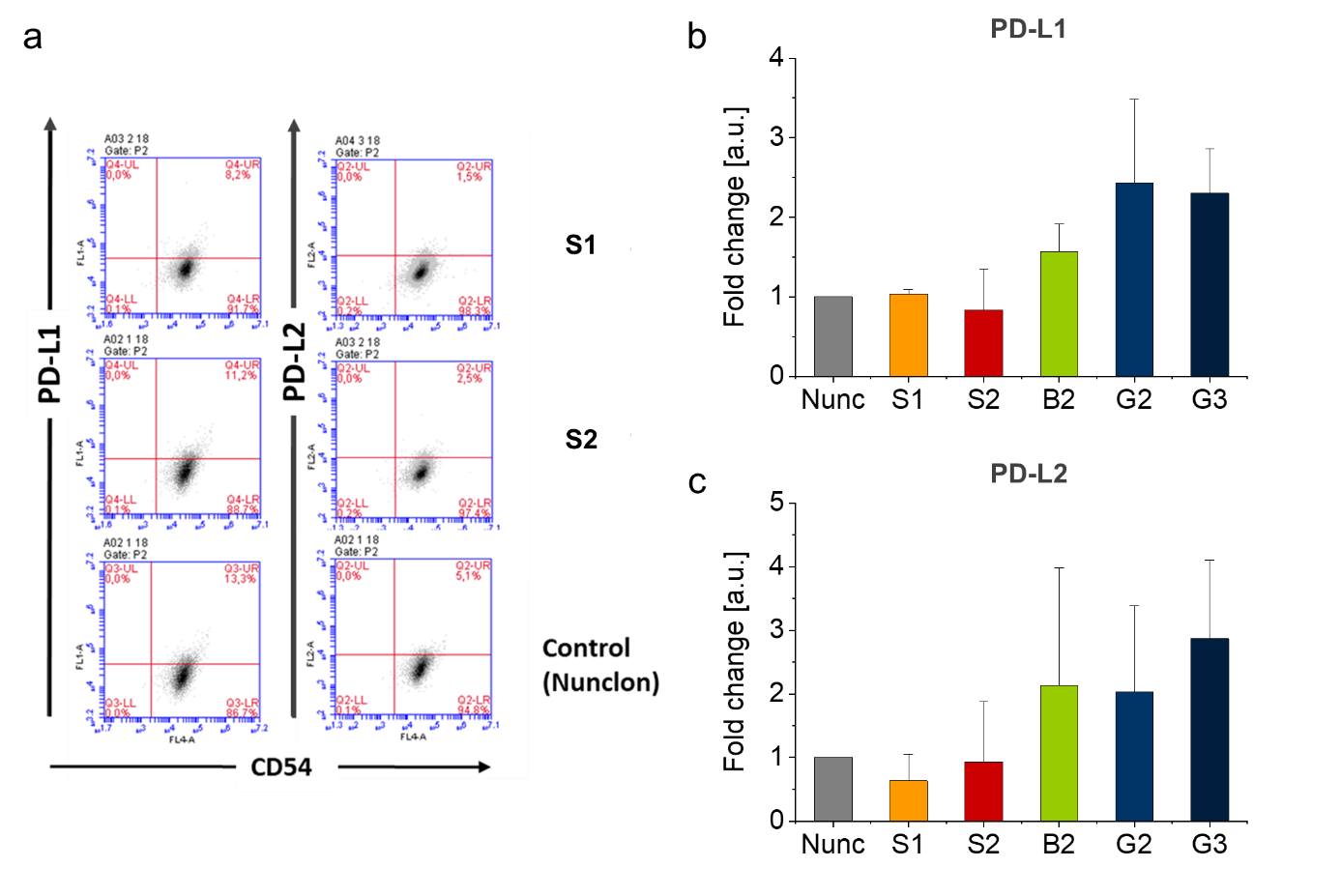


**Fig. S10.** Expression of immune-control associated molecules on CD54 positive moDCs cultured in the presence of soluble PGEs **S1** and **S2** at a concentration of 10 µg mL^-1^ and on **B2** brush as well as **G2** and **G3** gel coatings determined and analyzed by flow cytometry from n = 3 healthy donors on day 6 of culture. Flow cytometry dot plot analysis of PD-L1 and PD-L2 from one representative healthy donor (a) and fold change analysis of PD-L1 (b) and PD-L2 (c) expression from a mean of n = 3 donors against Nunc TCPS control substrates.


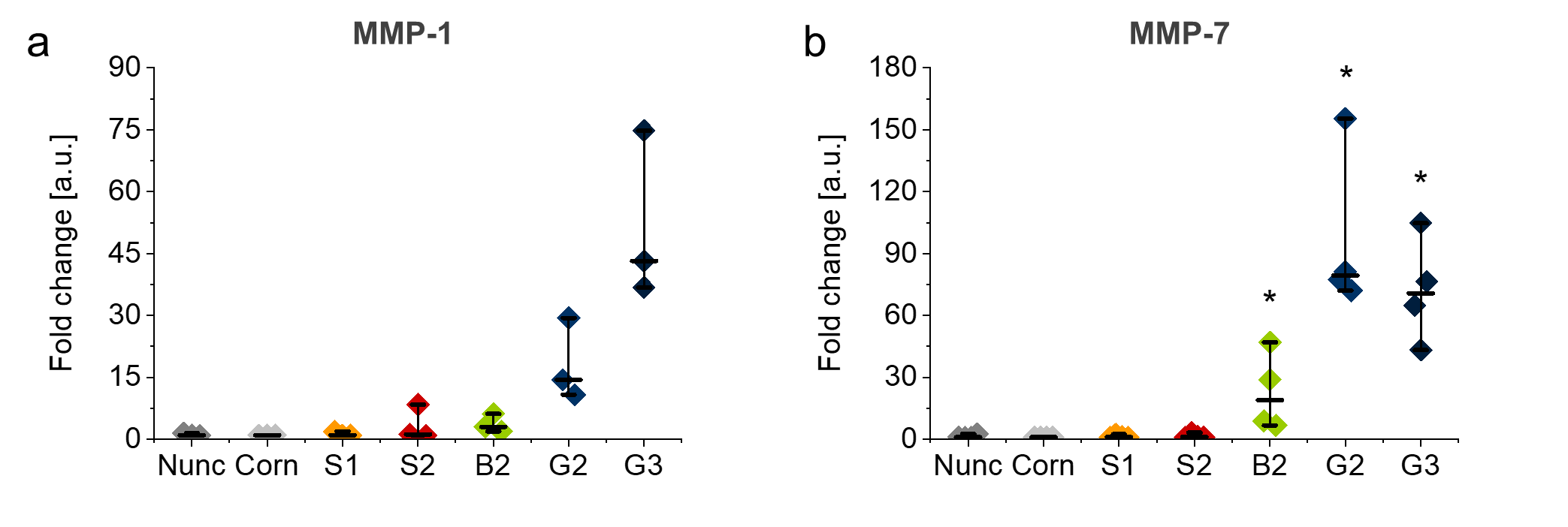


**Fig. S11.** Release of matrix metalloproteinase (MMP)-1 (a) and MMP-7 (b) by moDCs cultured in the presence of soluble PGEs **S1** and **S2** at a concentration of 10 µg mL^-1^ and on **B2** brush as well as **G2** and **G3** gel coatings and on Nunc and Corning (Corn) TCPS controls for 4 days. Protein levels were analyzed by ELISA in supernatants of moDCs generated from primary PBMCs of n = 3 (a) and n = 4 (b) healthy donors. Differences in MMP-1 and MMP-7 values were analyzed for statistical significance and compared to Nunc controls using the non-parametric Mann-Whitney-U test. Values of *p* < 0.05 were considered significant (*: *p* < 0.05).

**References**

[1] D.D. Stöbener, M. Uckert, J.L. Cuellar-Camacho, A. Hoppensack, M. Weinhart, Ultrathin poly(glycidyl ether) coatings on polystyrene for temperature-triggered human dermal fibroblast sheet fabrication, ACS Biomater. Sci. Eng. 3 (2017) 2155-2165. [https://doi.org/10.1021/acsbiomaterials.7b00270](about:blank)

[2] D.D. Stöbener, M. Weinhart, Thermoresponsive poly(glycidyl ether) brush coatings on various tissue culture substrates—how block copolymer design and substrate material govern self-assembly and phase transition, Polymers 12 (2020) 1899. [https://doi.org/10.3390/polym12091899](about:blank)

[3] D.D. Stöbener, M. Weinhart, On the foundation of thermal “switching”: The culture substrate governs the phase transition mechanism of thermoresponsive brushes and their performance in cell sheet fabrication, Acta Biomater. 136 (2021) 243-253. [https://doi.org/10.1016/j.actbio.2021.09.012](about:blank)

[4] S. Heinen, S. Rackow, J.L. Cuellar-Camacho, I.S. Donskyi, W.E.S. Unger, M. Weinhart, Transfer of functional thermoresponsive poly(glycidyl ether) coatings for cell sheet fabrication from gold to glass surfaces, J. Mater. Chem. B 6 (2018) 1489-1500. [https://doi.org/10.1039/C7TB03263C](about:blank)

[5] S. Heinen, S. Rackow, A. Schäfer, M. Weinhart, A perfect match: Fast and truly random copolymerization of glycidyl ether monomers to thermoresponsive copolymers, Macromolecules 50 (2017) 44-53. [https://doi.org/10.1021/acs.macromol.6b01904](about:blank)

[6] D.D. Stöbener, A. Hoppensack, J. Scholz, M. Weinhart, Endothelial, smooth muscle and fibroblast cell sheet fabrication from self-assembled thermoresponsive poly(glycidyl ether) brushes, Soft Matter 14 (2018) 8333-8343. [https://doi.org/10.1039/C8SM01099D](about:blank)

[7] J.L. Hutter, J. Bechhoefer, Calibration of atomic-force microscope tips, Rev. Sci. Instrum. 64 (1993) 1868-1873. [https://doi.org/10.1063/1.1143970](about:blank)

[8] H.-J. Butt, M. Jaschke, Calculation of thermal noise in atomic force microscopy, Nanotechnology 6 (1995) 1-7. [https://doi.org/10.1088/0957-4484/6/1/001](about:blank)
